# Crosstalk and ultrasensitivity in protein degradation pathways

**DOI:** 10.1371/journal.pcbi.1008492

**Published:** 2020-12-28

**Authors:** Abhishek Mallela, Maulik K. Nariya, Eric J. Deeds

**Affiliations:** 1 Department of Mathematics, University of California Davis, Davis, California, United States of America; 2 Laboratory of Systems Pharmacology, Harvard Medical School, Boston, Massachusetts, United States of America; 3 Department of Integrative Biology and Physiology, University of California, Los Angeles, Los Angeles, California, United States of America; 4 Institute for Quantitative and Computational Biosciences, University of California, Los Angeles, Los Angeles, California, United States of America; King’s College London, UNITED KINGDOM

## Abstract

Protein turnover is vital to cellular homeostasis. Many proteins are degraded efficiently only after they have been post-translationally “tagged” with a polyubiquitin chain. Ubiquitylation is a form of Post-Translational Modification (PTM): addition of a ubiquitin to the chain is catalyzed by E3 ligases, and removal of ubiquitin is catalyzed by a De-UBiquitylating enzyme (DUB). Nearly four decades ago, Goldbeter and Koshland discovered that reversible PTM cycles function like on-off switches when the substrates are at saturating concentrations. Although this finding has had profound implications for the understanding of switch-like behavior in biochemical networks, the general behavior of PTM cycles subject to synthesis and degradation has not been studied. Using a mathematical modeling approach, we found that simply introducing protein turnover to a standard modification cycle has profound effects, including significantly reducing the switch-like nature of the response. Our findings suggest that many classic results on PTM cycles may not hold *in vivo* where protein turnover is ubiquitous. We also found that proteins sharing an E3 ligase can have closely related changes in their expression levels. These results imply that it may be difficult to interpret experimental results obtained from either overexpressing or knocking down protein levels, since changes in protein expression can be coupled via E3 ligase crosstalk. Understanding crosstalk and competition for E3 ligases will be key in ultimately developing a global picture of protein homeostasis.

## Author summary

Previous work has shown that substrates of Post-Translational Modification (PTM) cycles can have coupled responses if those substrates share enzymes. This implies that modifications leading to substrate degradation (e.g. ubiquitylation by an E3 ligase) could introduce coupling in concentrations of substrates sharing a ligase. Using mathematical models, we found adding protein turnover to a PTM cycle diminishes both sensitivity and ultrasensitivity, particularly in models admitting long ubiquitin chains. We also found that proteins sharing an E3 ligase can indeed have coupled changes in both expression and sensitivity to signals. These results imply that accounting for crosstalk in protein degradation networks is crucial for the interpretation of results from a wide variety of common experimental perturbations to living systems.

## Introduction

All proteins undergo some form of turnover. For instance, proteins can become damaged via deamidation or some other process and must be degraded in order to prevent unfolding and aggregation. Turnover is also important in signaling and the regulation of protein function. A classic example is the degradation of *IκB* proteins, which bind the NF-*κB* protein complex and sequester it in the cytoplasm. During response to different stimuli, *IκB*s are phosphorylated by *IκB* kinases, ubiquitylated, and tagged for degradation, which allows NF-*κB* to translocate to the nucleus [1]. In the cell, synthesis and degradation (i.e. protein turnover) act in concert to maintain an appropriate concentration of active protein (i.e. protein homeostasis). Given the centrality of protein turnover to all cellular processes, it is not surprising that dysregulation of protein homeostasis has been implicated in a vast array of neurodegenerative diseases and cancers [2,3]. In eukaryotes, degradation is often achieved through the ubiquitin-proteasome system, where proteins are tagged with polyubiquitin chains that are recognized by the proteasome, ultimately leading to protein degradation [4]. Polyubiquitylation represents a form of post-translational modification (PTM) cycle where ubiquitin subunits are covalently linked to substrates by E3 ligases and removed by deubiquitylating (DUB) enzymes [5].

Over 35 years ago, Goldbeter and Koshland studied the general properties of a PTM cycle comprised of a modifying and demodifying enzyme. They found that reversible cycles of protein modification, such as a kinase enzyme adding a phosphoryl group and a phosphatase enzyme removing it, work like on-off switches when the enzymes are saturated [6]. This phenomenon, known as “0^th^-order ultrasensitivity”, has had profound implications for understanding how biochemical networks can exhibit switch-like behavior. Despite decades of progress in understanding 0^th^-order ultrasensitivity and other aspects of PTM function [7–12], to date there have been few attempts to systematically characterize the general behavior of PTM cycles that drive protein degradation.

The only exception to this has been the study of ubiquitylation in the context of cell cycle oscillations and bistability [13,14]. While these studies have provided key insights about cell cycle control, they have not investigated how ubiquitylation controls the steady-state expression levels of proteins not involved in the cell cycle. It has also been shown that adding protein synthesis and degradation to models of gene expression and cell signaling can have dramatic effects on system dynamics, but the detailed impact of turnover on PTM cycles remains unclear [15–17].

In addition to a general lack of understanding of the influence of protein homeostasis on PTM cycle behavior, we recently discovered that substrates in such cycles can have coupled steady-state responses if those substrates share modification/demodification enzymes. In particular, if one substrate is at saturating levels, or if the substrates collectively saturate the enzymes, then all substrates of that pair will respond in a coupled, switch-like manner [18–20]. This implies that modification leading to substrate degradation (e.g. ubiquitylation by an E3 ligase) could introduce coupling in the concentrations of substrates sharing a ligase. Interestingly, Mather and colleagues have shown that substrate concentrations can be coupled through saturation of the downstream degradation machinery [21,22]. It is currently unclear, though, whether such coupling can arise due to “crosstalk” in the upstream mechanisms that tag proteins for degradation.

In this work, we used a set of mathematical models to show that perturbing a standard PTM cycle by simply adding synthesis and degradation has profound effects on the response of the system. Specifically, we found that the sensitivity of the system to incoming signals and the ultrasensitivity of the response are dramatically muted when the substrate is at saturating concentrations. When the modification in question drives protein degradation at a higher rate, these effects are even more pronounced. Furthermore, more realistic models allowing for long ubiquitin chains exhibit qualitatively similar behavior to the case with a single modification state, but with further decreases in sensitivity and ultrasensitivity. These findings are robust to changes in the specific mechanisms utilized by the E3 and DUB enzymes. Interestingly, we found that distinct modes of enzyme saturation (i.e. increasing substrate production rate vs. decreasing the Michaelis constant of the enzyme) also generate different substrate responses. This indicates that many classic results on PTM cycles, including the extremely ultrasensitive response they exhibit when the substrates are at saturating concentrations, may not hold *in vivo* where protein turnover is inevitable. We also found that proteins sharing an E3 ligase can indeed have closely related expression profiles. Moreover, the sensitivity of protein concentration to changes in E3 activity for any given protein is largely dependent upon the total expression level of the other proteins. This suggests that it may be difficult to interpret experimental results obtained from either overexpressing or reducing protein concentrations, since changes in protein expression can be coupled via E3 ligase crosstalk. Further experimental characterization of E3-ligase/DUB enzyme/substrate relationships will thus be vital to developing a global understanding of protein regulation within the cell.

## Results

### Competition among E3 ligases

As mentioned in the introduction, shared E3 ligases have the potential to induce coupling in substrate responses. It is currently unclear, however, how widespread such “crosstalk” among E3 ligases might be. We searched the E3Net database [23] for statistics of E3-substrate interactions in human cells. For sake of comparison, we also obtained E3-specific statistics from the hUbiquitome database [24]. As of this writing, the total number of E3 ligases documented in E3Net was 415 and the total number of their substrates was 873, making the average ‘substrate load’ (substrate-to-ligase ratio) 2.10. Similarly, there are a total of 138 ligases and 279 substrates annotated in the hUbiquitome database, yielding a comparable ratio of 2.02. Thus, on average, most E3 ligases will ubiquitylate around two substrates.

**Fig 1:**
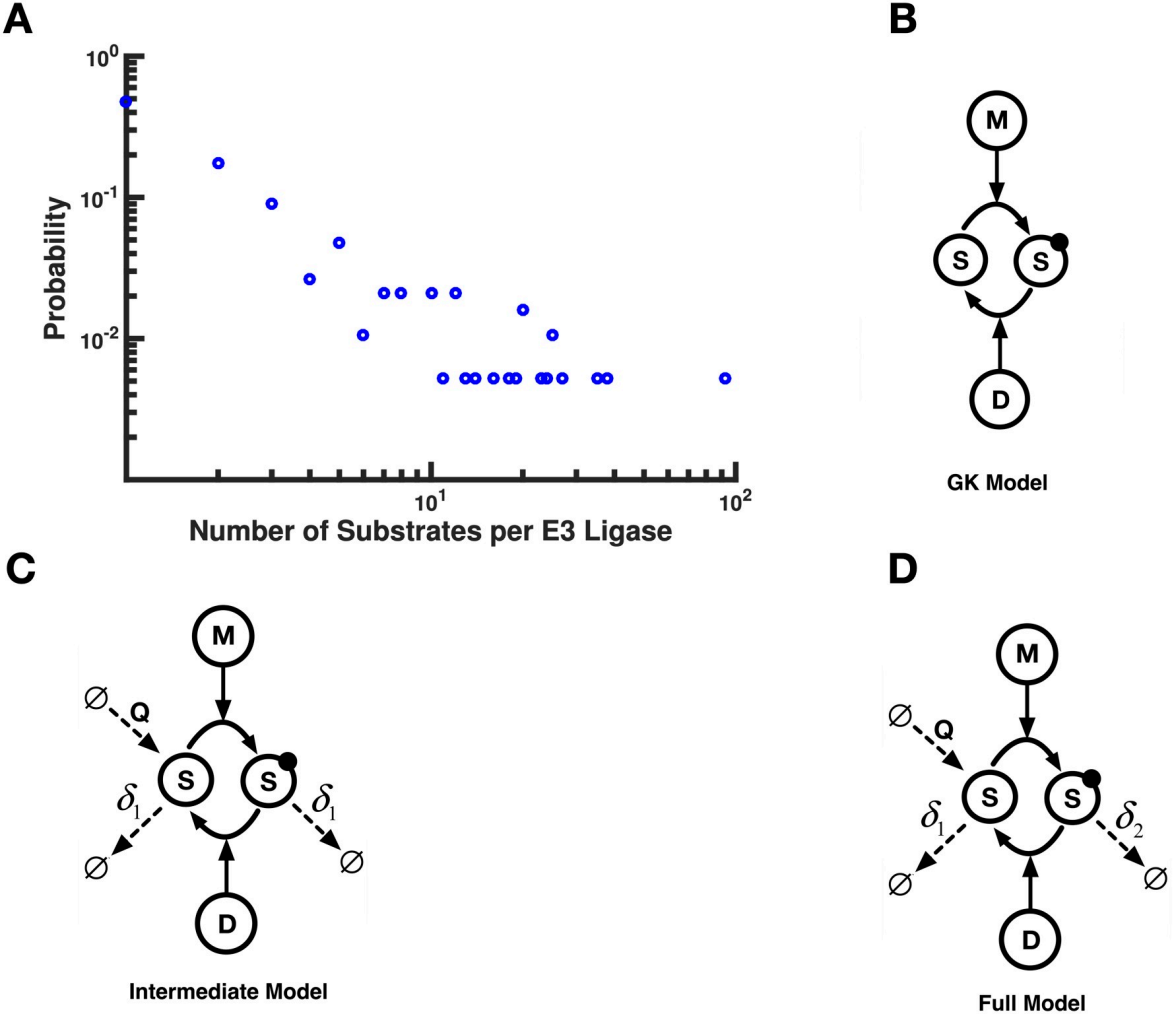
Crosstalk among E3 ligases & schematic diagrams of single-substrate models. **(A)** Probability distribution (on log-log scale) of E3 ligase-substrate specificity as recorded in the E3Net database. The average “substrate load” on a given E3 ligase is 2.1. In Panels B-D, the general representation of the canonical Goldbeter-Koshland (GK) loop is shown, with protein turnover included. **(B)** The case of the traditional GK loop, with no synthesis and degradation; **(C)** The “Intermediate” model, which introduces synthesis (at a rate *Q* > 0) and degradation (at a rate *δ*_1_ > 0); **(D)** The “Full” model, which includes synthesis (*Q* > 0) and to different degradation rates: *δ*_1_ for the unmodified substrate and *δ*_2_ for the modified substrate. Since the modification in this model is meant to represent a tag that drives degradation, the rate of degradation for the modified substrate is higher than that of the unmofied substrate (i.e. *δ*_2_ > *δ*_1_ > 0). Here “M” denotes modifying enzyme and “D” denotes demodifying enzyme. Modified substrate is indicated by the dark, filled circle.

In addition to providing the numbers of ligases and substrates, E3Net also captures information on specific E3-substrate interactions. We found that 54% of the E3 ligases in the database have no substrates listed; however, of the remaining E3 ligases, 52% have at least 2 substrates and 11% have more than 10 substrates (Fig 1A). Also, the maximum number of substrates for any ligase is 92. Given that the database is incomplete, it is likely that these numbers represent significant underestimates of E3 ligase crosstalk. Regardless, the phenomenon of E3 ligases acting on multiple substrates is likely widespread, and little is presently known about what influence crosstalk might have on the responses of these substrates to changes in E3 ligase activity.

### Adding synthesis and degradation to a PTM cycle

Even though E3 ligases generally attach long ubiquitin chains to their substrates [4], in order to simplify the problem to an analytically tractable form, we first considered a case with just a single modification state. Because ubiquitylation actively effects protein degradation, any investigation of the interplay between substrates competing for a protein and post-translational modifications (PTMs) leading to degradation must account for protein turnover. We thus focused first on studying how synthesis and degradation influence the behavior of the standard Goldbeter-Koshland loop (Fig 1B).

The first model, which we call the ‘Intermediate’ model, involves one substrate that can exist in two forms: modified and unmodified, denoted by S* and *S* respectively (Fig 1C). In this model, the modification (e.g. phosphorylation) does not lead to higher rates of degradation, so the modified and unmodified substrates are *both* degraded at the same first order rate *δ*_1_. Unmodified substrate is also synthesized at a constant rate *Q*. There is a modification enzyme *M* that catalyzes the addition of the PTM in question, and a demodification enzyme *D* that catalyzes removal of the modification. Since these are enzymes, they form enzyme-substrate complexes: *D* forms a complex with S* and *M* with *S*. To simplify the model, neither *M* nor *D* are subject to synthesis and degradation. We assume that the enzyme-substrate complexes are subject to “degradation” at the same rate *δ*_1_, but degradation in this case simply removes the substrate, essentially freeing the enzyme (note that relaxation of this assumption has no impact on our results, see S1 Text, Sec. 1).

The enzymatic reaction scheme corresponding to this model can be used to obtain a system of ordinary differential equations (ODEs) with the binding, dissociation, and catalysis steps treated explicitly (full details are in S1 Text, Sec. 1.1). We have denoted the kinetic rates of complex formation, complex dissociation, and catalysis by *k*_*x*,*y*_, where *x* represents the reaction step and *y* represents the enzyme—modifying (M) or demodifying (D) (S1 Text, Sec. 1.1). For example, *k*_*cat*,*D*_ denotes the catalytic rate of the reaction catalyzed by the demodifying enzyme.

Traditional analyses of post-translational modification cycles (i.e. the GK loop) have examined the response of molar fraction of modified protein at steady-state (S* ≡ [S*]/[*S*]_*T*_, where [*S*]_*T*_ = [*S*] + [S*] + [*MS*] + [DS*]) to changes in the input parameter $r\equiv \frac{k_{cat,M}{\left[M\right]}_{T}}{k_{cat,D}{\left[D\right]}_{T}}=\frac{V_{max,M}}{V_{max,D}}$, which quantifies the activity of the *M* enzyme relative to that of the *D* enzyme [6,18]. We varied *r* by simply changing [*M*] and numerically integrated the system to extract the steady-state solutions for unmodified and modified substrate ([*S*] and [S*]) at each value of *r*. Note that, for the Intermediate model, [*S*]_*T*_ = *Q*/*δ*_1_, regardless of the values of other parameters (S1 Text, Sec. 1.3). Here, we focus on the case where the Michaelis constants of the enzymes are equal (*K*_*M*,*M*_ = *K*_*M*,*D*_). We should note that our models only consider the standard Michaelis-Menten case where enzyme concentrations are significantly lower than substrate concentrations. Relaxing this assumption could have an influence on the behavior of the models when the concentration of the enzymes is similar to that of the substrates. That being said, analysis of such a scenario is beyond the scope of this work, and we leave exploration of that case to the future [25].

**Fig 2:**
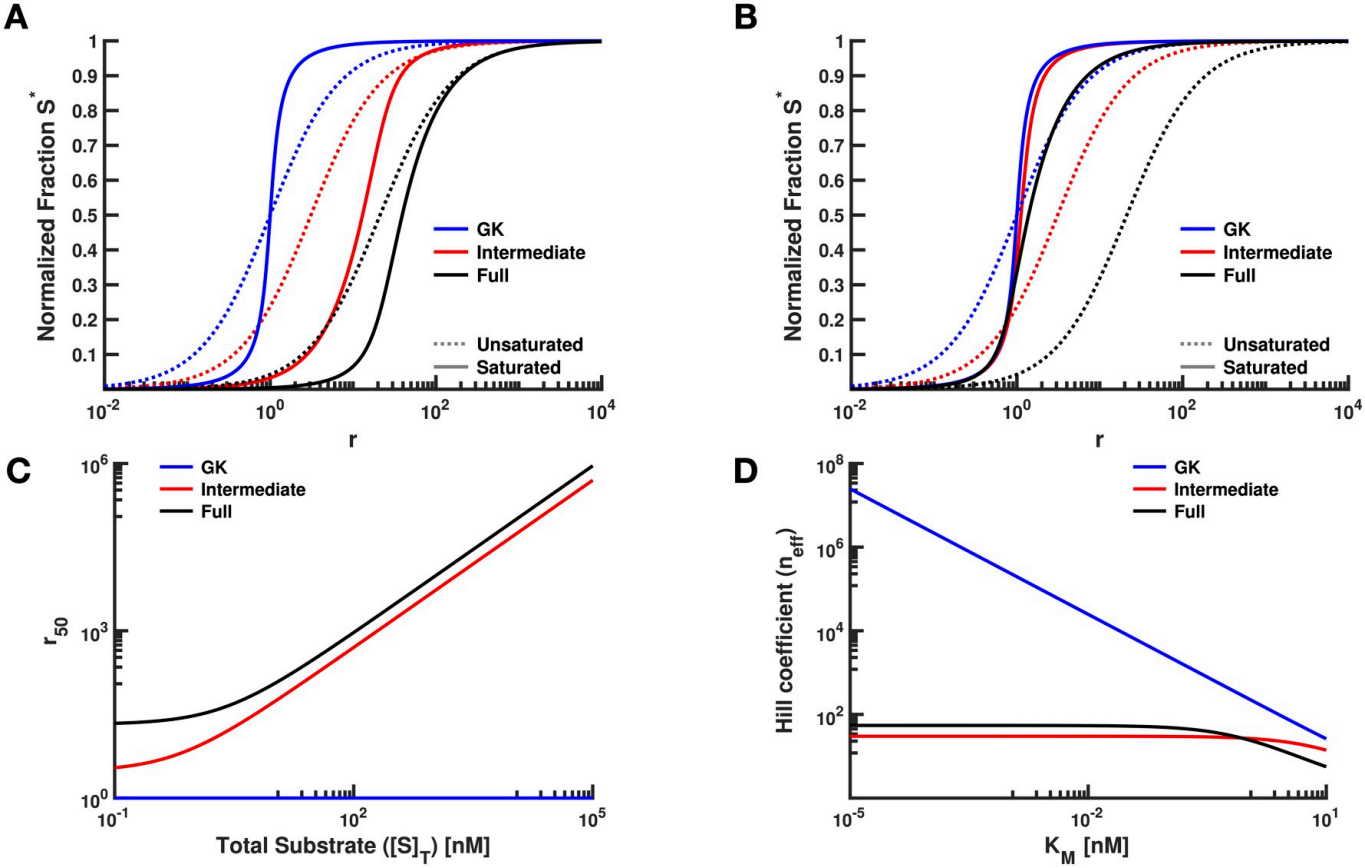
Effects on various single-substrate models of varying *Q* or *K*_*M*_ as the measure of enzyme saturation. **(A)** Modulating the rate of protein synthesis (*Q*) results in dramatic reduction of both sensitivity of the system to incoming signals and ultrasensitivity of the response, in the regime where the enzymes are saturated. This is indicated by the rightward shift of the *r*_50_ and the reduction in the Hill coefficient (*n*_*eff*_) from GK to Full. Note the logarithmic axis used for *r*. **(B)** Varying the Michaelis-Menten constant (*K*_*M*_) results in a smaller reduction of *r*_50_ and *n*_*eff*_, as compared to varying *Q*, in the regime of saturated enzymes. In Panels A-B, dashed lines and solid lines correspond to the unsaturated and saturated cases, respectively. Also, for all of the cases in these panels, [*D*]_*T*_ = 10^−1^ nM and [*M*]_*T*_ is varied in order to vary *r*. In Panel A, [*S*]_*T*_ = 10^3^ nM for the unsaturated cases and [*S*]_*T*_ = 10^5^ nM for the saturated cases. In Panel B, [*S*]_*T*_ = 10^3^ nM for all cases. **(C)** Increasing [*S*]_*T*_ does not change the *r*_50_ for the GK model, but the reduction in sensitivity is highly pronounced for the Intermediate model, and even more so for the Full model. Axes are on a log scale. **(D)** Increasing *K*_*M*_ results in a dramatic reduction of *n*_*eff*_ for the GK model. For systems that incorporate protein turnover (i.e. the Intermediate and Full models), the effect of reduction occurs for sufficiently large *K*_*M*_.

One key feature of GK loops is their capacity to exhibit 0^th^-order ultrasensitivity, which manifests as a switch-like transition in [S*] vs. *r* when the modification and demodification enzymes are saturated (i.e. [*S*]_*T*_ ≫ *K*_*M*_) [6,9,18,25]. To explore this phenomenon in the Intermediate model, we initially increased *Q* to change saturation levels, since [*S*]_*T*_ = *Q*/*δ*_1_. In order to conduct these simulations, we chose a set of reasonable values for the kinetic rate constants in the model (Table 1). In particular, *k*_*cat*_’s and *K*_*M*_’s were taken from experimentally observed ranges (S1 Text, Sec. 1.4, [26]) and *δ*_1_ was set based on the average observed half-life for proteins in living human cells [27]. The value for *δ*_1_ is also very similar to the shortest observed protein half-life in mouse C2C12 cells [20,28]. As can be seen from Fig 2A, there are dramatic differences between a GK loop and the Intermediate model upon saturation. For instance, defining *r*_50_ as the *r*-value when [S*] is half-maximal (i.e. S* = 0.5), we see that the curve of S* vs. *r* shifts to the right, indicating a higher *r*_50_. Secondly, the ultrasensitivity (i.e. the effective Hill coefficient *n*_*eff*_) of the system under the Intermediate model is reduced. Since the Intermediate model is simply a GK loop with synthesis and degradation (*Q* and *δ*_1_, compare Fig 1B and 1C) added, these results indicate that adding synthesis and degradation to a PTM can have a dramatic effect on 0^th^-order ultrasensitivity.

**Table 1:** Parameter values used for Fig 2A and 2B. Fig 2A: chosen on the basis of $\delta_{1}=\frac{\text{log}\;\left(2\right)}{10\;hours}$, which is the average reported protein half-life in human cells; Fig 2B: *K*_*M*_ = 10^1^ × [*S*]_*T*_ (unsaturated) and *K*_*M*_ = 10^−1^ × [*S*]_*T*_ (saturated).

Parameter	Fig 2A	Fig 2B	Units
*Q*(unsat.)	2.00 × 10^−2^	2.00 × 10^−2^	[nM] · [s]^−1^
*Q*(sat.)	2.00	2.00 × 10^−2^	[nM] · [s]^−1^
*k*_+_(unsat.)	1.00 × 10^−4^	1.00 × 10^−4^	[nM · s]^−1^
*k*_+_(sat.)	1.00 × 10^−4^	1.00 × 10^−2^	[nM · s]^−1^
*k*_	1.00 × 10^−3^	1.00 × 10^−3^	[s]^−1^
*k*_*cat*_	9.99 × 10^−1^	9.99 × 10^−1^	[s]^−1^
*δ*_1_	2.00 × 10^−5^	2.00 × 10^−5^	[s]^−1^
*δ*_2_	2.00 × 10^−4^	2.00 × 10^−4^	[s]^−1^

While increasing the expression level of *S* (e.g. increasing *Q*) is a natural way to achieve saturation, one can also saturate the enzymes by decreasing *K*_*M*_, keeping *Q* fixed. In a standard GK loop, varying [*S*]_*T*_ and varying *K*_*M*_ are mathematically equivalent; in the Intermediate model, however, the effects of decreasing *K*_*M*_ (with a lower bound of 100 nM for experimentally observed *K*_*M*_’s, S1 Text, Sec. 1.4) are dramatically different from the effects of increasing *Q*. In particular, the changes in *r*_50_ and *n*_*eff*_ are negligible (Fig 2B).

The findings above were obtained for just a single set of parameters, and this raises the question of whether or not our observations are robust to parameter variation. Since this model is relatively simple, we were able to characterize this parameter dependence analytically by solving the system of ODEs at steady state. We obtained the following equation relating *r*_50_ to *Q* and *K*_*M*_ when corresponding kinetic rate parameters for the modifying and demodifying enzymes are identical (S1 Text, Sec. 1.6):

$$r_{50}=(1+\frac{\delta_{1}}{k_{cat,D}})+(\frac{1}{2k_{cat,D}\cdot {\left[D\right]}_{T}})Q+(\frac{\delta_{1}}{k_{cat,D}\cdot {\left[D\right]}_{T}})K_{M}$$

Considering the endpoints of the plots in Fig 2C and the equation above, it is clear that as the rate of substrate production is made arbitrarily large, the *r*_50_ grows without bound. Thus the system described by the Intermediate model *always* becomes less and less sensitive to incoming signals as *Q* increases, regardless of the value of the other parameters. However, when we make *K*_*M*_ as small as possible with all other parameters fixed (i.e. *K*_*M*_ → 0), we see that $r_{50}\to 1+\frac{1}{k_{cat,D}}(\delta_{1}+\frac{Q}{2\cdot {\left[D\right]}_{T}})$, which is a constant. Note that this constant value is nevertheless always larger than *r*_50_ = 1 for the GK model, independent of the choices of the other parameters (S1 Text, Sec. 1.6).

In a similar fashion we can analyze the effective Hill coefficient (*n*_*eff*_) for the Intermediate model. Note that the S* vs. *r* curves do not precisely follow the form of a Hill function; as such, we use the standard definition $n_{eff}=\text{log}\;\left(81\right)/\text{log}\;\left(\frac{r_{90}}{r_{10}}\right)$ [6,29]. Our analytical results establish a lower bound on *n*_*eff*_ for the Intermediate model (which we will refer to as *n*_*eff*_(I)), indicating that the Intermediate model always exhibits positive cooperativity (i.e. *n*_*eff*_(I) > 1, S1 Text, Sec. 1.7). As with *r*_50_, we also find that varying saturation levels by changing total substrate concentration (i.e. changing *Q*) vs. changing *K*_*M*_ results in opposing effects on *n*_*eff*_ (Fig 2D). While *n*_*eff*_(GK) grows without bound as saturation increases, *n*_*eff*_(I) increases only modestly and is generally smaller by several orders of magnitude. For instance, when *Q* is increased, *n*_*eff*_(I) evaluates to exactly 2, regardless of the values of the other parameters.

The above results clearly demonstrate that changing saturation by varying *Q* and varying *K*_*M*_ have very different consequences for the steady-state response of the PTM cycle in the Intermediate model. Specifically, increasing saturation of the enzymes by increasing total substrate levels (in other words, by increasing *Q*) results in responses that are much less *sensitive* (i.e. *r*_50_ increases, requiring greater *M* activity in order to achieve an appreciable response) and much less *ultrasensitive* (i.e. lower *n*_*eff*_) than would be predicted by a traditional GK loop (Fig 1B) [6,7]. The intuitive reason behind this has to do with the ultimate source of extreme ultrasensitivity in the original GK model. In that case, there is neither synthesis nor degradation, so steady state can only be achieved when the flux of S* production by *M* precisely matches the flux of *S* production by *D*. If substrate is at high concentration ([*S*]_*T*_ ≫ *K*_*M*_), then both enzymes can theoretically operate at their *V*_*max*_. If *V*_*max*,*M*_ ≠ *V*_*max*,*D*_ (i.e. *r* ≠ 1), this means that one of the enzymes can operate faster than the other. Say that *V*_*max*,*M*_ > *V*_*max*,*D*_. In that scenario, the only way for the enzymatic fluxes to be balanced is for the *M* enzyme to operate *below* its *V*_*max*_. In other words, the *M* enzyme must convert so much of the substrate from the *S* to the S* state that it becomes unsaturated, meaning [*S*] ≪ *K*_*M*_. Since we are in a condition with [*S*]_*T*_ ≫ *K*_*M*_, this directly implies that [*S*]/*K*_*M*_ ≪ 1, and as a result S* will be close to 1. When the *D* enzyme has a higher *V*_*max*_ (*r* < 1), then the situation is reversed, and S* will be close to 0 at steady state. This leads to a very switch-like behavior in S* as *r* is increased in the original GK loop (Fig 2).

In the Intermediate model, however, additional fluxes have been added, namely synthesis and degradation. In particular, steady state is not achieved when the flux of S* production by *M* matches the flux of de-modification by *D*, but rather when the flux of S* production matches the flux of de-modification *plus* the flux of degradation of S*. In this model, degradation is first-order, so it can never be saturated; as [S*] increases, so will the flux of degradation. As more and more substrate is added to the system, the velocity of substrate modification must thus increase in order to match this increasing flux of degradation, if [S*] is to be relatively high at steady-state. Under such conditions, the *M* enzyme will be operating at *V*_*max*_, and so the only way to increase the rate of modification of the substrate is to increase *V*_*max*,*M*_, thus increasing *r*. This leads to the increase in *r*_50_ as the steady-state levels of [*S*]_*T*_ increase. Similarly, since degradation helps balance *S* modification at steady-state, there is no longer an extreme switch-like response in S* vs. *r* (Fig 2). Because varying *K*_*M*_ changes saturation without altering the steady-state level of substrate, the effect of changing *K*_*M*_ is very different from varying *Q* in the Intermediate model.

We should note that since the value of *K*_*M*_ depends on the underlying rate constants for the enzyme-substrate interaction, it is unlikely to vary on short, cellular time scales. In other words, it is hard to imagine how a cell would increase the saturation of an enzyme by dynamically lowering the *K*_*M*_ of one of its enzymes during the course of a response to an environmental fluctuation. On evolutionary time scales, however, an enzyme’s *K*_*M*_ can change, subject to reasonable physical and biological constraints. So, it is more likely that saturation will change by changing the production rate *Q in vivo*. Certainly, experimental manipulation of saturation generally occurs through changes in protein expression (e.g. by “overexpressing” the protein, which would correspond to increasing *Q* in this model). Thus, even for cases like phosphorylation where the PTM does not directly influence degradation rate, the steady-state responses of PTM cycles *in vivo* may thus be quite different from the standard predictions that have been made in the absence of any consideration of protein turnover [7–12].

### Driving protein degradation: The “Full” model

While the results described above hold for any PTM cycle subject to turnover, we are ultimately interested in PTMs like ubiquitylation that drive protein degradation. This corresponds in our case to *δ*_2_ > *δ*_1_, which we term the “Full” model. For the purposes of display, we kept *δ*_1_ close to the average degradation rate of human proteins and set *δ*_2_ close to the fastest degradation rate observed in human cells (i.e. *δ*_1_ = 2 × 10^−5^*s*^−1^ and *δ*_2_ = 2 × 10^−4^*s*^−1^) [27]. The point here is to focus our analysis on a case where the PTM drives rapid degradation, as is often thought to be the case when a substrate is ubiquitylated *in vivo* [4].

We first considered how changes in E3 ligase activity relative to DUB activity would influence the modification state of the substrate. To facilitate comparison with the Intermediate and GK models, we initially focused our analysis on the steady-state level of substrate modification, S*. We found that transitions in S* are even less sensitive to incoming signals in the full model, as compared to the Intermediate model (Fig 2A and 2B). Indeed, the *r*_50_ for the full model is always greater than that for the Intermediate model as *Q* is increased (Fig 2C), and we have shown analytically that this is true for any reasonable set of kinetic parameters (S1 Text, Sec. 1.6). Intuitively, in the Full model, the modified substrate has a higher degradation rate than the unmodified state, so that the system needs to be driven harder towards the modified state (i.e. by increasing *r*) in order to appreciably increase its concentration at steady state. Interestingly, although *n*_*eff*_(I) is always less than *n*_*eff*_(GK) as discussed above, we find that *n*_*eff*_(Full) is less than *n*_*eff*_(I) only for very small *Q*. For instance, when *Q* is increased without bound *n*_*eff*_(Full) evaluates to exactly $\text{log}\;\left(81\right)/[\text{log}\;\left(9\right)+\text{log}\;\left(\frac{9\delta_{1}+\delta_{2}}{9\delta_{2}+\delta_{1}}\right)]$, or approximately 7 in our case, which is larger than lim_*Q*→∞_ *n*_*eff*_ (I) = 2.

**Fig 3:**
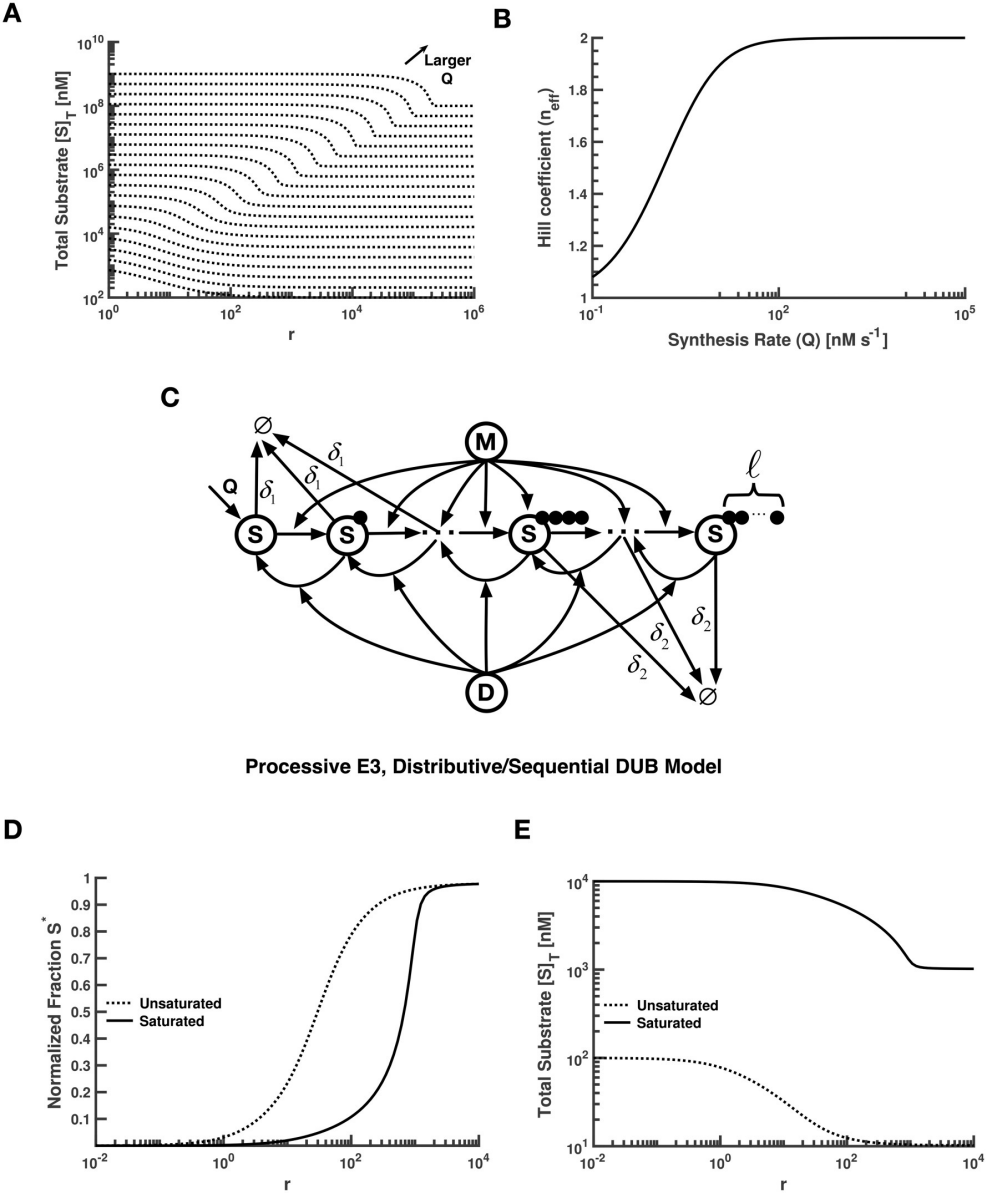
Effects of varying *Q* on the *r*_50_ and *n*_*eff*_ in the Full model and its representative analog for multiple modification states. **(A)** The shift in *r*_50_ for the transition in total substrate at increasing values of *Q* is clearly evident here. Each dashed curve indicates a different value for *Q*. The maximum and minimum values of [*S*]_*T*_ for each curve is *Q*/*δ*_1_ and *Q*/*δ*_2_, respectively. **(B)** The effective Hill coefficient *n*_*eff*_ is relatively unaffected by increase in protein synthesis rate in the Full model. **(C)** Schematic diagram for one substrate with multiple modification states. Shown here is the model corresponding to the Processive E3, Distributive/Sequential DUB case. Each of the first three units is degraded at the rate *δ*_1_, which is smaller than the rate *δ*_2_ for the remaining units. The maximum length of the polyubiquitin chain is denoted by *ℓ*. **(D)** In the Processive E3, Distributive/Sequential DUB model, much more E3 ligase activity is necessary for a maximal response in the saturated regime. **(E)** Compared to Panel (A), the rightward shift is more pronounced in the presence of polyubiquitin chains.

Since E3 ligase activity drives higher levels of protein degradation in the full model, changes in the *r* parameter will change not only [S*] but also the total concentration of substrate ([*S*]_*T*_). Perhaps not surprisingly, we found that [*S*]_*T*_ also exhibits an ultrasensitive transition in *r*. As with the transitions in [S*] discussed above, there is a rightward shift in *r*_50_ for the [*S*]_*T*_ vs. *r* curve as *Q* is increased in the full model (S1 Text, Sec. 1.8). This phenomenon can generate interesting behaviors, as shown in Fig 3A. Suppose that we systematically increase the expression level of the protein while keeping the concentrations of the modifying/demodifying enzymes constant, which corresponds to a constant *r* in this model. As *Q* increases, the *r*_50_ of the curve increases from a point less than this constant value of *r* to a point greater than *r*. This leads to nonlinear changes in total substrate as Q increases (see below). The [*S*]_*T*_ vs. *r* curve has a number of other similarities to the S* vs. *r* curve; for instance, we see that the effective Hill coefficient for total substrate in the full model does not change significantly when *Q* is increased, and never exceeds a value of 2 (Fig 3B).

So, in the Full model, as in the Intermediate model, increasing substrate levels by increasing *Q* results in a decrease in sensitivity (increase in *r*_50_) and limited ultrasensitivity in the response, regardless of whether we consider the response parameter to be S* or [*S*]_*T*_ (which is likely the more relevant response parameter *in vivo*). The similarities in this case all arise from the fact that, in these models, steady state can only be achieved when the flux of the *M* enzyme matches the sum total of the fluxes due to the *D* enzyme and the degradation process (note Eq. 8 in S1 Text, Sec. 1.3). This gives rise to a fundamentally different phenomenology than is observed in the standard GK loop (Figs 2 and 3).

### Adding multiple modification states to the full model

While the full model is suggestive, it abstracts a number of details of the biological systems that control protein homeostasis. For instance, E3 ligases, rather than adding just a single ubiquitin to their substrates, instead tend to attach polyubiquitin chains of varying lengths. Most of the available literature suggests that this ubiquitin chain needs to be at least four units long in order for the proteasome to efficiently recognize and degrade the substrate [13,30,31]. While the specific details of both the requisite length of the chain and the E3/DUB enzyme mechanisms are still being elucidated, it is fairly clear that a single ubiquitin is not sufficient to drive effective degradation by the proteasome.

To capture these effects in our models, we surveyed available literature and found that multiple enzymatic mechanisms have been proposed for both E3 ligases and DUB enzymes [13,30–38]. E3 ligases may be “processive”, in the sense that the ligase adds an ubiquitin unit to the polyubiquitin chain at each catalytic step and stays attached to the substrate while multiple ubiquitins are added sequentially. Alternatively, they may be “distributive”, meaning that the ligase disassociates from the substrate at the end of each catalytic reaction. In the one case that has been extensively studied experimentally, a form of E3 called a RING ligase works with the E2 Cdc34 to build polyubiquitin chains on substrates in a processive manner [37]. Of course, this does not mean that other E3 ligases might not display distributive kinetics. Regarding the DUB enzyme counterpart, 3 such enzymes have been found in 26S proteasomes: Rpn11, Usp14, and Uch37 [32–34,38]. Rpn11 functions by truncating at the base of the chain (in a distributive manner), whereas Usp14 and Uch37 serve primarily to trim the ubiquitin chains sequentially (in a processive manner). Interestingly, more than one DUB might act on a given chain [32].

Although there are experimentally characterized examples for several of these possible mechanisms, little is actually known about how widespread each mechanism may be in nature. We thus employed an exhaustive approach, examining all combinations of the enzyme mechanisms and creating models of those scenarios. In our initial analysis, the parameter values we used for the distributive cases correspond to the values in the previous section (i.e. Single Substrate, Single Modification State). However, we used parameter values directly from literature for the processive cases [37].

While we have analyzed all 6 possible combinations of enzyme mechanisms (see S1 Text, Sec. 2), given the available experimental data [37], we focus our discussion in the main text on a reasonable and representative case (Processive E3 and Distributive/Sequential DUB) from this set of models. We have depicted this scheme in Fig 3C. As mentioned above, current consensus indicates that a polyubiquitin chain typically requires at least four ubiquitin units to be efficiently degraded by the proteasome [4]. We thus assumed that each of the first three modification states (0–3 ubiquitins) is degraded at a uniform rate, *δ*_1_, which is smaller than the corresponding (higher) rate *δ*_2_ for each of the remaining states (4 or more ubiquitins). In theory, the ubiquitin chain could reach an infinite length, though of course in practice the action of DUBs and degradation will limit the largest chain typically observed in the system. Denoting this maximum length of the ubiquitin chain by *ℓ*, we enumerated the chemical reaction networks for all of the possible mechanistic scenarios described above (S1 Text, Sec. 2). Due to the inherent complexity of the models, we could not obtain closed-form analytical solutions, and thus focused on numerical simulations.

Recall that in the full model, which has a single modification state, we found a significant reduction in both sensitivity and ultrasensitivity of the transition in S* when compared to the Intermediate model. To compare our more complex model with the full model, we defined S* for the case with multiple modification states as follows: $S^{*}\equiv \sum_{k=4}^{l}\left[S^{\left(k\right)}\right]/{\left[S\right]}_{T}$, where *k* indexes the substrate modification state. To choose a reasonable value for *ℓ*, we systematically increased this parameter and found a threshold value such that changes in *r*_50_ and *n*_eff_ were negligible beyond that threshold. Using this approach, we chose a value of 50 for *ℓ* heuristically by visual inspection. In Fig 3D and 3E, we see that the inclusion of ubiquitin chains magnifies the aforementioned effects in both the S* vs. *r* and [*S*]_*T*_ vs. *r* curves. Specifically, much more E3 ligase activity is necessary to achieve a maximal response in saturated regimes.

As described above, numerical integration of the ODEs for all of the multiple modification state models necessitated the definition of a maximum possible length for the ubiquitin chain (*ℓ*). To determine if this truncation has any influence on the results, we developed an “agent-based” stochastic simulation (see S1 Text, Sec. 2.8). Rather than representing the system as a set of concentrations for each species, in these simulations we consider a finite population of explicit substrate “agents.” Each of these substrate molecules has associated with it the length of its ubiquitin chain, which ranges from 0 (no ubiquitin) to the largest number we can represent as a floating point number, allowing the length to be essentially arbitrary. Because of their discrete representation of the substrates, these simulations are stochastic, and we parameterized these simulations so that the stochastic rates correspond exactly to the parameters we used for our deterministic simulations (see S1 Text, Sec. 2.8) [39–42]. We found excellent agreement between the deterministic results and the agent-based simulations for all of the enzyme mechanisms we considered, suggesting that truncating the system at *ℓ* = 50 yields a reasonable approximation.

Interestingly, all of the models that we examined, arising from the various mechanisms proposed for the E3 ligase and DUB enzymes, generated similar qualitative behavior (S1 Text, Sec. 2.8–2.9). Thus, while the quantitative details of the response (e.g. the value of *r*_50_ and *n*_*eff*_) vary somewhat between the models, our general findings are largely invariant with respect to the catalytic mechanisms utilized by the E3 ligase and DUB enzymes [4]. The results presented above, however, all focus on a single set of parameters; while these parameters are reasonable, it is not clear how reasonable variation in the parameters might affect our findings. We focused on how variation in the *K*_*M*_ and *δ* parameters might influence our findings, sampling these parameters from log-uniform distributions across two orders of magnitude centered on the set of parameters considered above. Again, while changes in these parameters influence the quantitative details of the response, the qualitative behaviors of these models are all consistent with our original observations. In particular, we found that saturation increases the value of *r*_50_ in the transition of both S* and [*S*]_*T*_ in all cases (S1 Text, Sec. 2.8–2.9).

As in the case of the Full model, the fundamental reason we observe similar behavior in these multiple modification state models, regardless of the specific enzyme mechanism, has to do with the fact that all of those details simply modify the relationship between *M* enzyme activity and the flux of degradation of the substrate. In other words, regardless of the kinetic scheme, there are two populations of molecules: those degraded more slowly (*δ*_1_) and those degraded more rapidly (*δ*_2_). In order to reach a steady state, the flux of the *M* enzyme that produces these rapidly degraded species must match the flux of degradation plus the flux of de-modification by the *D* enzyme. As substrate concentration increases, more *M* activity is required to do this, leading to the increase in *r*_50_ and the overall lower level of ultransensitivity that we observe. Thus, while modification of both the parameters and the enzymatic scheme change some of the quantitative features of the response, the fundamental behavior is similar in all cases.

One interesting thing to note here is that PTM systems with multiple modifications can readily lead to multistability, with distinct stable steady-states corresponding to different distributions of the concentration of the various modification states [43–44]. We did not observe this kind of phenomenon in any of our models, most likely because we assumed that the rates of the enzymatic reactions do not depend on the modification state itself (see S1 Text, Sec. 2.1–2.6). Relaxation of this assumption may lead to multistability in ubiquitylation states, which could have important consequences for the function of the ubiquitin-proteasome system. To our knowledge, there has been no experimental observation of multistability in ubiquitylation, likely due in part to the fact that no one has to date designed an experiment aimed at testing this idea explicitly. We leave the exploration of the potential for multistability in these systems to future work.

### Adding multiple substrates to the full model

**Fig 4:**
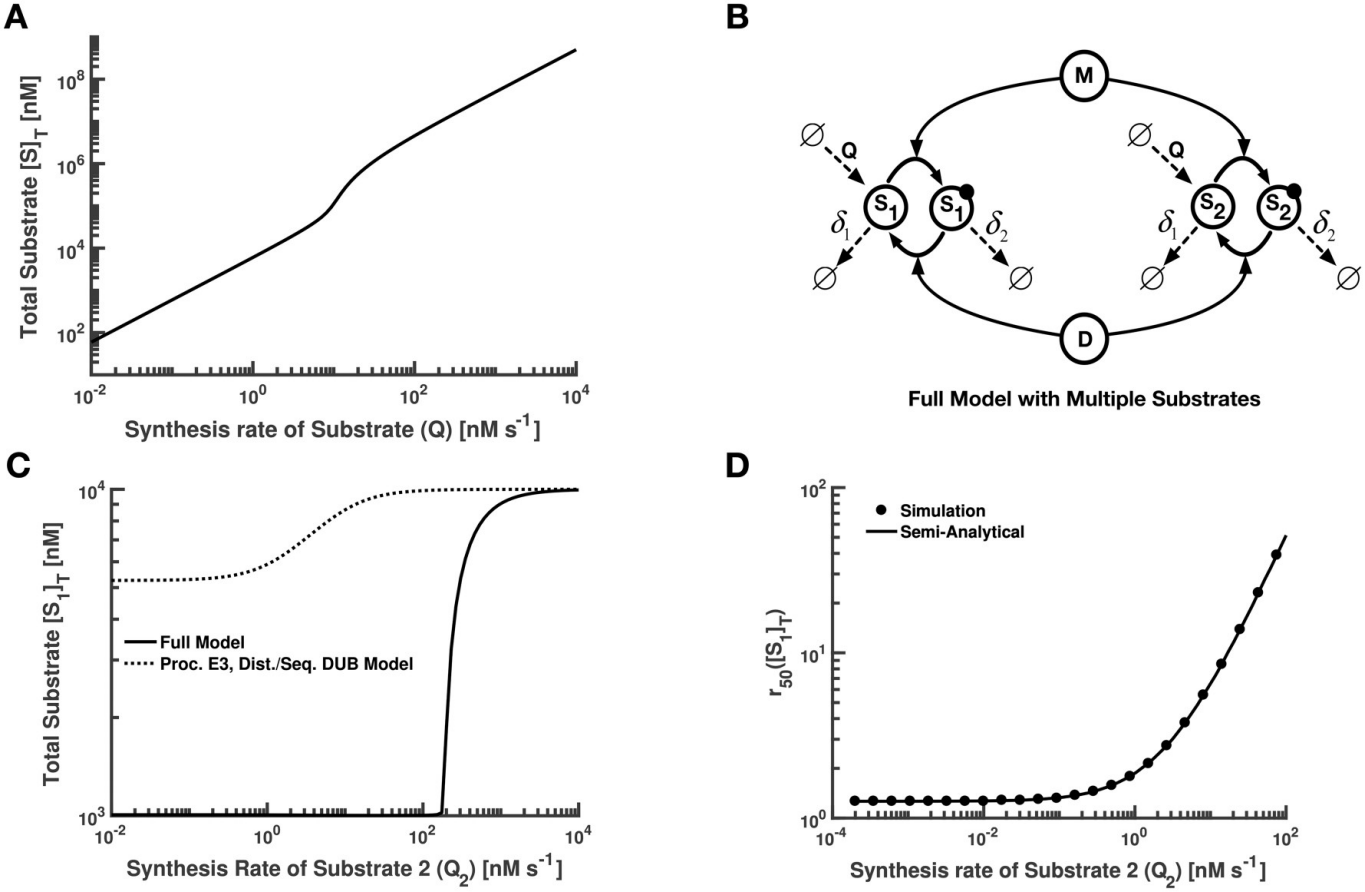
Effects of protein overexpression on total protein in single-substrate and multiple-substrate models. **(A)** There is a non-linear transition in the [*S*]_*T*_ vs. *Q* curve when *Q* is comparable in magnitude with the measure of saturation (in this case, [*M*]). The slope of the curve approaches 1 on either side of the transition, which is consistent with the maximum and minimum values of [*S*]_*T*_. **(B)** Schematic diagram for multiple substrates with one modification state each. Shown here is the model corresponding to two substrates, for simplicity. Here “M” denotes modifying enzyme and “D” denotes demodifying enzyme. Modified substrate is indicated by the dark, filled circle. **(C)** Plot of total concentration of first substrate vs. synthesis rate of the second substrate, for the multiple-substrate analogs of the Full model and the representative multiple modification state model. Axes are on a log scale. **(D)** Sensitivity to signal for first substrate vs. synthesis rate of the second substrate, for the multiple-substrate analogue of the Full model. The semi-analytical curve was obtained by substituting values for $S_{2}^{*}$ obtained empirically from simulation into *α*_2_ in the analytical expression for *r*_50_ ([*S*_1_]_*T*_). Axes are on a log scale.

As mentioned above (Fig 3A), there is an increase in *r*_50_ for the [*S*]_*T*_ vs. *r* curve as *Q* is increased in the full model. As a consequence, increasing *Q* while keeping *r* fixed at a value of 100 results in the curve seen in Fig 4A. For low values of *Q*, the transition in *r*_50_ occurs before this fixed *r*-value, so [*S*]_*T*_ ≈ *Q*/*δ*_2_; for large *Q*, the transition in *r*_50_ occurs after this fixed *r*-value, so [*S*]_*T*_ ≈ *Q*/*δ*_1_. For intermediate *Q*, however, there is a distinct transition between these two regimes. In other words, as *Q* increases, the system will naturally transition between a point “after” the transition in [*S*]_*T*_ to one “before” the transition in [*S*]_*T*_, due to the impact that saturation has on *r*_50_. The result in Fig 4A implies that if two substrates share an E3/DUB enzyme pair, the *r*_50_’s of the transitions in total substrate concentrations for these two proteins will be coupled. In other words, overexpression of one substrate could shift the total saturation, and thus the *r*_50_, of all the substrates coupled to that E3/DUB pair.

To test this, we introduced more than one substrate in the context of the full model. For the sake of display, we have taken the total number of substrates *N* in our model to be 2. As shown in Fig 4B, each E3 ligase and DUB now acts on two substrates with one modification state per substrate. The set of ODEs describing the model is given in S1 Text, Sec. 3. In contrast to the case of just one substrate, here we are interested in capturing the response of ${\left[S_{1}\right]}_{T}=[S_{1}]+[S_{1}^{*}]$ to changes in the synthesis rate of the second substrate, denoted by *Q*_2_. In Fig 4C, we see that increasing *Q*_2_ yields an increase in [*S*_1_]_*T*_ for the Full model, as expected. The general behavior is similar in the Processive E3 and Distributive/Sequential DUB model, with the transition in [*S*_1_]_*T*_ occurring at lower values of *Q*_2_.

Interestingly, *r*_50_([*S*_1_]_*T*_) also depends on *Q*_2_ (Fig 4D). Specifically, when *Q*_2_ is large enough in the Full model, *r*_50_ increases linearly with respect to *Q*_2_. In a similar manner to Fig 4C, a lower value of *Q*_2_ is sufficient to obtain a linear increase in *r*_50_ in the presence of multiple modification states. Note the excellent agreement in the Full model between the *r*_50_ values extracted empirically from simulation output and the analytical expression for *r*_50_([*S*_1_]_*T*_) (Fig 4D and S1 Text, Sec. 3.3). In fact, when we make *Q*_2_ arbitrarily large, we obtain:

$$r_{50}({\left[S_{1}\right]}_{T})=[\frac{\delta_{2}}{V_{max,\;D}}((\frac{1}{\alpha_{2}}-1)\delta_{1}+\delta_{2})]Q_{2}$$

where *α*_2_ is the molar fraction of modified *S*_2_ at steady-state when [*S*_1_]_*T*_ is half-maximal. In other words, as the concentration of the second substrate is increased, more and more activity of the E3 ligase (i.e. *M*) is needed to drive the transition in *S*_1_ concentration. Interestingly, all of these results can be readily generalized for any number of substrates, independent of substrate identity (S1 Text, Sec. 3.2–3.3). Thus, crosstalk in PTMs can lead to coupling of not only modification states [18,19], but also of overall protein levels.

## Discussion

**Fig 5:**
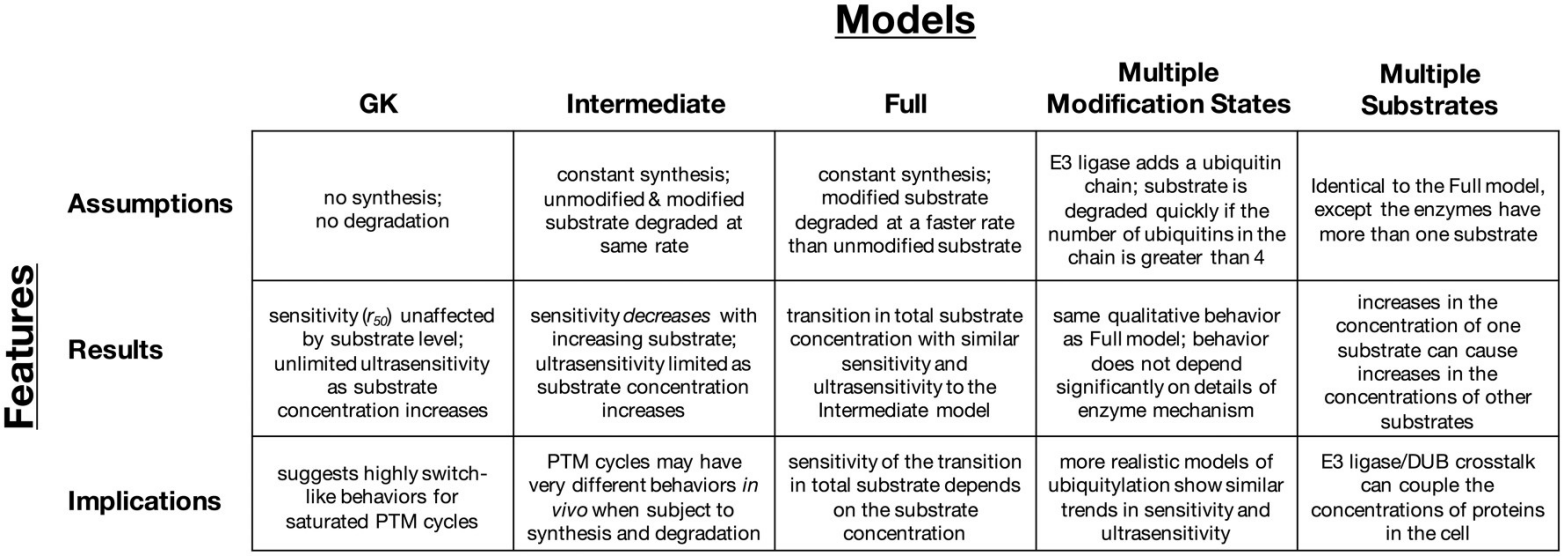
A summary of the key features and results for each model considered here.

It has been over 35 years since Goldbeter and Koshland discovered the phenomenon of 0^th^-order ultrasensitivity. Since then, there has been extensive characterization of PTM cycles with 0^th^-order ultrasensitivity, both experimentally [45–47] and computationally [18,20,25,48,49]. Until now, however, the properties of PTM cycles that drive protein degradation have not been studied in a systematic way. Using a mathematical modeling framework, we found that adding synthesis and degradation to a PTM cycle suppresses both sensitivity to signal and ultrasensitivity of the response, even when the PTM in question does not serve as a signal for protein degradation (Fig 5). Thus switch-like behaviors *in vivo* may or may not be the consequence of 0^th^-order ultrasensitivity, depending on the stability of the protein substrate. Although there are exceptions [15–17], most models of signaling networks ignore protein turnover [50,51]. Our findings indicate that incorporating turnover, especially turnover based on actual protein stabilities, is key to capturing the global PTM dynamics of signaling systems.

Interestingly, we found the general trend of decreasing sensitivity and ultrasensitivity holds for PTMs that drive protein degradation, even when accounting for many of the complicated mechanisms that describe polyubiquitylation by E3 ligases and deubiquitylation by DUB enzymes (Fig 5 and S1 Text, Sec. 2). By adding E3 ligase crosstalk, we demonstrated that overexpressing one protein can elevate the concentration of another, and can also reduce the sensitivity of other proteins to incoming signals that would drive their degradation (Fig 4C and 4D). In other words, if one protein is overexpressed, it becomes more difficult to degrade any of its counterparts sharing the same E3/DUB enzyme pair.

Although there is some data available about the specificity of E3 ligases [34,52,53], this information is very far from complete. Consider the highly common experimental scenario where a primary aim is to characterize the function of a protein by manipulating its expression level. Our findings indicate that the interpretation of overexpression data in eukaryotic cells may be very difficult because some of the observed phenotypic or molecular effects could be directly due to the higher concentration of the protein that was expressed, but other effects could be due to E3 ligase coupling (Fig 4C). Additional complications could appear due to the change in sensitivity to the shared E3 ligases for other substrates in the system (Fig 4D). For instance, if a protein is being actively regulated by its E3 ligase and a degradation signal appears, then a high concentration of other proteins in the system would potentially inhibit the signal. This could have unforeseen large-scale effects on the overall system.

A global picture of E3-ligase/DUB enzyme specificity will thus likely be essential to comprehending the regulation of protein levels within cells. This will allow us to begin determining how to isolate direct effects of changes in protein expression levels from indirect effects. Equally necessary are mathematical or computational models of signaling dynamics, gene regulatory networks, and other cellular processes that describe the interplay between PTMs that do not lead to degradation and those that drive degradation. Incorporating the coupled dynamics of protein levels into our understanding of cell signaling and cellular physiology thus represents a grand challenge for both experimental and computational systems biology.

## Materials and methods

### Experimental methods

Our model behaviors can be described deterministically by systems of ordinary differential equations (ODEs). Numerical integration of the systems was performed by the stiff solver ode15s in MATLAB. All analyses were performed at steady-state. In parallel, agent-based stochastic simulations of the systems [39–41] were conducted using custom-built software implemented in C++. Parameter values were chosen to ensure equivalence between the deterministic and stochastic systems. See the supporting information for full details regarding all of the models considered here.

## Supporting information

S1 Text This appendix contains further details about the models studied here, as well as additional mathematical and numerical results. (PDF)

## References

[1] Gasparian AV, Yao YJ, Kowalczyk D, Lyakh LA, Karseladze A, Slaga TJ, et al The role of IKK in constitutive activation of NF-kappaB transcription factor in prostate carcinoma cells. J Cell Sci. 2002 1 1;115(Pt 1):141–51. 11801732 10.1242/jcs.115.1.141

[2] Calamini B, Morimoto RI. Protein homeostasis as a therapeutic target for diseases of protein conformation. Curr Top Med Chem. 2012;12(22):2623–40. 10.2174/1568026611212220014 23339312 PMC3955168

[3] Powers ET, Morimoto RI, Dillin A, Kelly JW, Balch WE. Biological and chemical approaches to diseases of proteostasis deficiency. Annu Rev Biochem. 2009;78:959–91. 10.1146/annurev.biochem.052308.114844 19298183

[4] Finley D. Recognition and processing of ubiquitin-protein conjugates by the proteasome. Annu Rev Biochem. 2009;78:477–513. 10.1146/annurev.biochem.78.081507.101607 19489727 PMC3431160

[5] Komander D, Rape M. The ubiquitin code. Annu Rev Biochem. 2012;81:203–29. 10.1146/annurev-biochem-060310-170328 22524316

[6] Goldbeter A, Koshland DE. An amplified sensitivity arising from covalent modification in biological systems. Proc Natl Acad Sci U S A. 1981 11;78(11):6840–4. 10.1073/pnas.78.11.6840 6947258 PMC349147

[7] Goldbeter A, Koshland DE. Ultrasensitivity in biochemical systems controlled by covalent modification. Interplay between zero-order and multistep effects. J Biol Chem. 1984 12 10;259(23):14441–7. 6501300

[8] Xing J, Chen J. The Goldbeter-Koshland switch in the first-order region and its response to dynamic disorder. PLoS One. 2008 5 14;3(5):e2140 10.1371/journal.pone.0002140 18478088 PMC2374878

[9] Xu Y, Gunawardena J. Realistic enzymology for post-translational modification: zero-order ultrasensitivity revisited. J Theor Biol. 2012 10 21;311:139–52. 10.1016/j.jtbi.2012.07.012 22828569 PMC3432734

[10] Martins BM, Swain PS. Ultrasensitivity in phosphorylation-dephosphorylation cycles with little substrate. PLoS Comput Biol. 2013;9(8):e1003175 10.1371/journal.pcbi.1003175 23950701 PMC3738489

[11] Zhang Q, Bhattacharya S, Andersen ME. Ultrasensitive response motifs: basic amplifiers in molecular signalling networks. Open Biol. 2013 4 24;3(4):130031 10.1098/rsob.130031 23615029 PMC3718334

[12] Ferrell JE, Ha SH. Ultrasensitivity part I: Michaelian responses and zero-order ultrasensitivity. Trends Biochem Sci. 2014 10;39(10):496–503. 10.1016/j.tibs.2014.08.003 25240485 PMC4214216

[13] Nguyen LK, Dobrzyński M, Fey D, Kholodenko BN. Polyubiquitin chain assembly and organization determine the dynamics of protein activation and degradation. Front Physiol. 2014;5:4 10.3389/fphys.2014.00004 24478717 PMC3901042

[14] Nguyen LK, Muñoz-García J, Maccario H, Ciechanover A, Kolch W, Kholodenko BN. Switches, excitable responses and oscillations in the Ring1B/Bmi1 ubiquitination system. PLoS Comput Biol. 2011 12;7(12):e1002317 10.1371/journal.pcbi.1002317 22194680 PMC3240587

[15] Loriaux PM, Hoffmann A. A framework for modeling the relationship between cellular steady-state and stimulus-responsiveness. Methods Cell Biol. 2012;110:81–109. 10.1016/B978-0-12-388403-9.00004-7 22482946 PMC5763568

[16] Loriaux PM, Hoffmann A. A protein turnover signaling motif controls the stimulus-sensitivity of stress response pathways. PLoS Comput Biol. 2013;9(2):e1002932 10.1371/journal.pcbi.1002932 23468615 PMC3585401

[17] Loriaux PM, Tesler G, Hoffmann A. Characterizing the relationship between steady state and response using analytical expressions for the steady states of mass action models. PLoS Comput Biol. 2013;9(2):e1002901 10.1371/journal.pcbi.1002901 23509437 PMC3585464

[18] Rowland MA, Fontana W, Deeds EJ. Crosstalk and competition in signaling networks. Biophys J. 2012 12 5;103(11):2389–98. 10.1016/j.bpj.2012.10.006 23283238 PMC3514525

[19] Rowland MA, Deeds EJ. Crosstalk and the evolution of specificity in two-component signaling. Proc Natl Acad Sci U S A. 2014 4 15;111(15):5550–5. 10.1073/pnas.1317178111 24706803 PMC3992699

[20] Rowland MA, Harrison B, Deeds EJ. Phosphatase specificity and pathway insulation in signaling networks. Biophys J. 2015 2 17;108(4):986–96. 10.1016/j.bpj.2014.12.011 25692603 PMC4336360

[21] Ogle CT, Mather WH. Proteolytic crosstalk in multi-protease networks. Phys Biol. 2016 4 4;13(2):025002 10.1088/1478-3975/13/2/025002 27042892

[22] Cookson NA, Mather WH, Danino T, Mondragón-Palomino O, Williams RJ, Tsimring LS, et al Queueing up for enzymatic processing: correlated signaling through coupled degradation. Mol Syst Biol. 2011 12 20;7:561 10.1038/msb.2011.94 22186735 PMC3737734

[23] Han Y, Lee H, Park JC, Yi GS. E3Net: a system for exploring E3-mediated regulatory networks of cellular functions. Mol Cell Proteomics. 2012 4;11(4):O111.014076. 10.1074/mcp.O111.014076 22199232 PMC3322580

[24] Du Y, Xu N, Lu M, Li T. hUbiquitome: a database of experimentally verified ubiquitination cascades in humans. Database (Oxford). 2011;2011:bar055. 10.1093/database/bar055 22134927 PMC3228279

[25] Gomez-Uribe C, Verghese GC, Mirny LA. Operating regimes of signaling cycles: statics, dynamics, and noise filtering. PLoS Comput Biol. 2007 12;3(12):e246 10.1371/journal.pcbi.0030246 18159939 PMC2230677

[26] Scheer M, Grote A, Chang A, Schomburg I, Munaretto C, Rother M, et al BRENDA, the enzyme information system in 2011. Nucleic Acids Res. 2011 1;39(Database issue):D670–6. 10.1093/nar/gkq1089 21062828 PMC3013686

[27] Eden E, Geva-Zatorsky N, Issaeva I, Cohen A, Dekel E, Danon T, et al Proteome half-life dynamics in living human cells. Science. 2011 2 11;331(6018):764–8. 10.1126/science.1199784 21233346

[28] Cambridge SB, Gnad F, Nguyen C, Bermejo JL, Krüger M, Mann M. Systems-wide proteomic analysis in mammalian cells reveals conserved, functional protein turnover. J Proteome Res. 2011 12 2;10(12):5275–84. 10.1021/pr101183k 22050367

[29] Dubitzky W, Wolkenhauer O, Cho K, Yokota H. Encyclopedia of Systems Biology. Springer Publishing Company, Incorporated; 2013.

[30] Proctor CJ, Tsirigotis M, Gray DA. An in silico model of the ubiquitin-proteasome system that incorporates normal homeostasis and age-related decline. BMC Syst Biol. 2007 3 21;1:17 10.1186/1752-0509-1-17 17408507 PMC1847462

[31] Kleiger G, Saha A, Lewis S, Kuhlman B, Deshaies RJ. Rapid E2-E3 assembly and disassembly enable processive ubiquitylation of cullin-RING ubiquitin ligase substrates. Cell. 2009 11 25;139(5):957–68. 10.1016/j.cell.2009.10.030 19945379 PMC2804849

[32] Lee MJ, Lee BH, Hanna J, King RW, Finley D. Trimming of ubiquitin chains by proteasome-associated deubiquitinating enzymes. Mol Cell Proteomics. 2011 5;10(5):R110.003871. 10.1074/mcp.R110.003871 20823120 PMC3098602

[33] Livneh I, Cohen-Kaplan V, Cohen-Rosenzweig C, Avni N, Ciechanover A. The life cycle of the 26S proteasome: from birth, through regulation and function, and onto its death. Cell Res. 2016 8;26(8):869–85. 10.1038/cr.2016.86 27444871 PMC4973335

[34] Lu Y, Lee BH, King RW, Finley D, Kirschner MW. Substrate degradation by the proteasome: a single-molecule kinetic analysis. Science. 2015 4 10;348(6231):1250834 10.1126/science.1250834 25859050 PMC4450770

[35] Metzger MB, Hristova VA, Weissman AM. HECT and RING finger families of E3 ubiquitin ligases at a glance. J Cell Sci. 2012 2 1;125(Pt 3):531–7. 10.1242/jcs.091777 22389392 PMC3381717

[36] Metzger MB, Pruneda JN, Klevit RE, Weissman AM. RING-type E3 ligases: master manipulators of E2 ubiquitin-conjugating enzymes and ubiquitination. Biochim Biophys Acta. 2014 1;1843(1):47–60. 10.1016/j.bbamcr.2013.05.026 23747565 PMC4109693

[37] Pierce NW, Kleiger G, Shan SO, Deshaies RJ. Detection of sequential polyubiquitylation on a millisecond timescale. Nature. 2009 12 3;462(7273):615–9. 10.1038/nature08595 19956254 PMC2791906

[38] Ventii KH, Wilkinson KD. Protein partners of deubiquitinating enzymes. Biochem J. 2008 9 1;414(2):161–75. 10.1042/BJ20080798 18687060 PMC2724835

[39] Feret J, Danos V, Krivine J, Harmer R, Fontana W. Internal coarse-graining of molecular systems. Proc Natl Acad Sci U S A. 2009 4 21;106(16):6453–8. 10.1073/pnas.0809908106 19346467 PMC2672529

[40] Deeds EJ, Krivine J, Feret J, Danos V, Fontana W. Combinatorial complexity and compositional drift in protein interaction networks. PLoS One. 2012;7(3):e32032 10.1371/journal.pone.0032032 22412851 PMC3297590

[41] Chylek LA, Harris LA, Tung CS, Faeder JR, Lopez CF, Hlavacek WS. Rule-based modeling: a computational approach for studying biomolecular site dynamics in cell signaling systems. Wiley Interdiscip Rev Syst Biol Med. 2014 Jan-Feb;6(1):13–36. 10.1002/wsbm.1245 24123887 PMC3947470

[42] Nariya MK, Israeli J, Shi JJ, Deeds EJ. Mathematical Model for Length Control by the Timing of Substrate Switching in the Type III Secretion System. PLoS Comput Biol. 2016 4;12(4):e1004851 10.1371/journal.pcbi.1004851 27078235 PMC4831731

[43] Markevich NI, Hoek JB, Kholodenko BN. Signaling switches and bistability arising from multisite phosphorylation in protein kinase cascades. J Cell Biol. 2004 2 2;164(3):353–9. 10.1083/jcb.200308060 14744999 PMC2172246

[44] Takahashi K, Tanase-Nicola S, ten Wolde PR. Spatio-temporal correlations can drastically change the response of a MAPK pathway. Proc Natl Acad Sci U S A. 2010 2 9;107(6):2473–8. 10.1073/pnas.0906885107 20133748 PMC2811204

[45] Hardie DG, Salt IP, Hawley SA, Davies SP. AMP-activated protein kinase: an ultrasensitive system for monitoring cellular energy charge. Biochem J. 1999 3 15;338 (Pt 3):717–22. 10051444 PMC1220108

[46] LaPorte DC, Koshland DE. Phosphorylation of isocitrate dehydrogenase as a demonstration of enhanced sensitivity in covalent regulation. Nature. 1983 9 22–28;305(5932):286–90. 10.1038/305286a0 6312317

[47] Meinke MH, Bishop JS, Edstrom RD. Zero-order ultrasensitivity in the regulation of glycogen phosphorylase. Proc Natl Acad Sci U S A. 1986 5;83(9):2865–8. 10.1073/pnas.83.9.2865 3458247 PMC323407

[48] Gunawardena J. Multisite protein phosphorylation makes a good threshold but can be a poor switch. Proc Natl Acad Sci U S A. 2005 10 11;102(41):14617–22. 10.1073/pnas.0507322102 16195377 PMC1253599

[49] Thomson M, Gunawardena J. Unlimited multistability in multisite phosphorylation systems. Nature. 2009 7 9;460(7252):274–7. 10.1038/nature08102 19536158 PMC2859978

[50] Albeck JG, Burke JM, Spencer SL, Lauffenburger DA, Sorger PK. Modeling a snap-action, variable-delay switch controlling extrinsic cell death. PLoS Biol. 2008 12 2;6(12):2831–52. 10.1371/journal.pbio.0060299 19053173 PMC2592357

[51] Suderman R, Deeds EJ. Machines vs. ensembles: effective MAPK signaling through heterogeneous sets of protein complexes. PLoS Comput Biol. 2013;9(10):e1003278 10.1371/journal.pcbi.1003278 24130475 PMC3794900

[52] Merbl Y, Refour P, Patel H, Springer M, Kirschner MW. Profiling of ubiquitin-like modifications reveals features of mitotic control. Cell. 2013 2 28;152(5):1160–72. 10.1016/j.cell.2013.02.007 23452859 PMC3711129

[53] Merbl Y, Kirschner MW. Large-scale detection of ubiquitination substrates using cell extracts and protein microarrays. Proc Natl Acad Sci U S A. 2009 2 24;106(8):2543–8. 10.1073/pnas.0812892106 19181856 PMC2633216

