# Supplementary material for: Crosstalk and ultrasensitivity in protein degradation pathways
Source: PLoS Comput Biol. 2020 Dec 28;16(12):e1008492. doi: 10.1371/journal.pcbi.1008492 (PMC7793289; doi:10.1371/journal.pcbi.1008492)
Supplement: S1 Text — (PDF) [file pcbi.1008492.s001.pdf]

Crosstalk and Ultrasensitivity in Protein Degradation Pathways

Supporting Information

Abhishek Mallela[1], Maulik K. Nariya[2] and Eric J. Deeds[3,4]

[1]Department of Mathematics, University of California Davis, Davis, CA, USA
[2]Laboratory of Systems Pharmacology, Harvard Medical School, Boston, MA, USA
[3]Department of Integrative Biology and Physiology, University of California Los Angeles, Los Angeles, CA, USA
[4]Institute for Quantitative and Computational Biosciences, University of California Los Angeles, Los Angeles, CA, USA

Email: Eric J. Deeds - deeds@ucla.edu;

Contents

1 Single Substrate, Single Modification State **4**

1.1 Equations . . . 4

1.1.1 ODEs . . . 4

1.1.2 Mass conservation . . . 5

1.2 Model definitions . . . 5

1.3 Preliminaries . . . 5

1.3.1 Steady-state . . . 5

1.3.2 Equivalence . . . 6

1.3.3 Total substrate . . . 6

1.3.4 Parameter Values . . . 6

1.4 Analytical expressions for r_{50} . . . 8

1.4.1 Intermediate Model . . . 8

1.4.2 Full Model . . . 8

1.5 Analytical expressions for n_{eff} . . . 9

1.6 Analysis of r_{50} in saturated regimes . . . 10

1.7 Analysis of n_{eff} . . . 10

1.8 Analytical expression for r_{50} of $[S]_T$ in Full model . . . 11

1.9 Analytical expression for n_{eff} of $[S]_T$ in Full model . . . 12

2 Single Substrate, Multiple Modification States 13

2.1 Model with Distributive E3 & Trunk DUB . . . 13

2.1.1 Enzymatic reaction scheme . . . 13

2.1.2 Parameter Values . . . 14

2.2 Model with Distributive E3 & Distributive/Sequential DUB . . . 15

2.2.1 Enzymatic reaction scheme . . . 15

2.2.2 Parameter Values . . . 15

2.3 Model with Distributive E3 & Processive/Sequential DUB . . . 16

2.3.1 Enzymatic reaction scheme . . . 16

2.3.2 Parameter Values . . . 17

2.4 Model with Processive E3 & Trunk DUB . . . 17

2.4.1 Enzymatic reaction scheme . . . 17

2.4.2 Parameter Values . . . 18

2.5 Model with Processive E3 & Distributive/Sequential DUB . . . 18

2.5.1 Enzymatic reaction scheme . . . 18

2.5.2 Parameter Values . . . 19

2.6 Model with Processive E3 & Processive/Sequential DUB . . . 20

2.6.1 Enzymatic reaction scheme . . . 20

2.6.2 Parameter Values . . . 20

2.7 Graphical results . . . 21

2.8 Stochastic simulations . . . 23

2.8.1 No truncation effect . . . 23

2.9 Robustness of results . . . 25

3 Multiple Substrates, Single Modification State 27

3.1 Equations . . . 27

3.1.1 Main . . . 27

3.1.2 Mass conservation . . . 27

3.2 Comments . . . 28

3.3 Analytical expression for $r_{50}([S_1]_T)$ . . . 28

4 Multiple Substrates, Multiple Modification States **29**

4.1 Model with Processive E3 & Distributive/Sequential DUB 29

5 Main Text: Parameter Values **30**

References **32**

1 Single Substrate, Single Modification State

We begin with a modification of the Goldbeter-Koshland loop that incorporates protein turnover. It is described by the following scheme of enzymatic reactions:

$$M + S \underset{k_{-,M}}{\overset{k_{+,M}}{\rightleftharpoons}} MS \xrightarrow{k_{cat,M}} M + S^*$$

$$D + S^* \underset{k_{-,D}}{\overset{k_{+,D}}{\rightleftharpoons}} DS^* \xrightarrow{k_{cat,D}} D + S$$

$$\emptyset \xrightarrow{Q} S$$

$$S \xrightarrow{\delta_1} \emptyset$$

$$S^* \xrightarrow{\delta_2} \emptyset$$

$$MS \xrightarrow{\delta_1} M$$

$$DS^* \xrightarrow{\delta_2} D$$

Here M and D represent any modifying and demodifying enzyme, respectively. Any substrate in the S state is degraded at a first-order rate δ_1, and any in the S^* state at a rate δ_2, with $\delta_2 > \delta_1$. Substrate is also synthesized at a constant rate Q, and all the synthesized substrates are in the S (unmodified) state. Note that degradation does not consume M or D.

(We find that relaxing this assumption only changes Equations (5) and (6), the ODEs that correspond to the concentration of the free M and D enzyme. Interestingly, these two equations are not used anywhere in the derivation. As a result, relaxing this assumption has no effect on our findings.)

It is straightforward to use the Law of Mass Action to formulate the corresponding set of ordinary differential equations (ODEs), as described below.

1.1 Equations

1.1.1 ODEs

$$\frac{d[S]}{dt} = Q - k_{+,M}[M][S] + k_{-,M}[MS] + k_{cat,D}[DS^*] - \delta_1[S] \tag{1}$$

$$\frac{d[S^*]}{dt} = -k_{+,D}[D][S^*] + k_{-,D}[DS^*] + k_{cat,M}[MS] - \delta_2[S^*] \tag{2}$$

$$\frac{d[MS]}{dt} = k_{+,M}[M][S] - (k_{-,M} + k_{cat,M} + \delta_1)[MS] \tag{3}$$

$$\frac{d[DS^*]}{dt} = k_{+,D}[D][S^*] - (k_{-,D} + k_{cat,D} + \delta_2)[DS^*] \tag{4}$$

$$\frac{d[M]}{dt} = (k_{-,M} + k_{cat,M} + \delta_1)[MS] - k_{+,M}[M][S] \tag{5}$$
$$\frac{d[D]}{dt} = (k_{-,D} + k_{cat,D} + \delta_2)[DS^*] - k_{+,D}[D][S^*] \tag{6}$$

1.1.2 Mass conservation

$$[M]_T = [M] + [MS]$$
$$[D]_T = [D] + [DS^*]$$
$$[S]_T = [S] + [S^*] + [MS] + [DS^*]$$

1.2 Model definitions

Using the formalism above, we can describe the Goldbeter-Koshland model as having no synthesis or degradation (i.e. with $Q = \delta_2 = \delta_1 = 0$). The Intermediate model has a positive synthesis rate and a uniform rate of degradation (i.e. with $Q > 0$ and $\delta_2 = \delta_1 > 0$.) Finally, the Full model also has a positive synthesis rate, but has different rates of degradation (i.e. $Q > 0$ and $\delta_2 > \delta_1 > 0$.)

1.3 Preliminaries

1.3.1 Steady-state

Let $K_{M,1} \equiv \frac{k_{-,M}+k_{cat,M}+\delta_1}{k_{+,M}} = K_{M,M} + \frac{\delta_1}{k_{+,M}}$ and $K_{M,2} \equiv \frac{k_{-,D}+k_{cat,D}+\delta_2}{k_{+,D}} = K_{M,D} + \frac{\delta_2}{k_{+,D}}$. We study the system at steady-state (i.e. setting the L.H.S. of eq. (1) - eq. (6) equal to zero). From the steady-state versions of eq. (3) and eq. (4), $[MS] = \frac{[M]\cdot[S]}{K_{M,M}}$ and $[DS^*] = \frac{[D]\cdot[S^*]}{K_{M,D}}$. Then

$$[M]_T = [M] + [MS] = [M]\left(1 + \frac{[S]}{K_{M,M}}\right) \implies [MS] = \frac{\left(\frac{[M]_T}{1+\frac{[S]}{K_{M,M}}}\right)[S]}{K_{M,M}} = \frac{[M]_T[S]}{K_{M,M} + [S]}$$

$$[D]_T = [D] + [DS^*] = [D]\left(1 + \frac{[S^*]}{K_{M,D}}\right) \implies [DS^*] = \frac{\left(\frac{[D]_T}{1+\frac{[S^*]}{K_{M,D}}}\right)[S^*]}{K_{M,D}} = \frac{[D]_T[S^*]}{K_{M,D} + [S^*]}$$

Adding eq. (1) and eq. (3) at steady-state yields

$$Q - \delta_1[S] + k_{cat,D}[DS^*] = (k_{cat,M} + \delta_1)[MS] \tag{7}$$

Adding eq. (2) and eq. (4) at steady-state gives

$$k_{cat,M}[MS] - \delta_2[S^*] = (k_{cat,D} + \delta_2)[DS^*] \tag{8}$$

1.3.2 Equivalence

In order to proceed with our analysis, we first establish that eq. (7) is equivalent to eq. (8). Note that adding eq. (1) - eq. (4) at steady-state yields

$$Q = \delta_1([S] + [MS]) + \delta_2([S^*] + [DS^*]) \tag{9}$$

Substituting eq. (9) in eq. (7) gives

$$\delta_1[MS] + \delta_2[S^*] + \delta_2[DS^*] + k_{cat,D}[DS^*] = (k_{cat,M} + \delta_1)[MS]$$

which is equivalent to eq. (8) upon simplification.

1.3.3 Total substrate

By the mass conservation equations, since $[M], [D] \geq 0$, we have $[MS] \leq [M]_T$ and $[DS^*] \leq [D]_T$. In the derivations that follow, we make the standard (Michaelis-Menten) assumption that total substrate is much larger than the concentration of either enzyme, so $[S]_T \approx [S] + [S^*]$. Note that eq. (9) implies $[S]_T = \frac{Q}{\delta_1}$ in the Intermediate model, since $\delta_1 = \delta_2$. For the full model, eq. (9) gives:

$$Q = \delta_1([S] + [MS]) + (\delta_1 + \delta_2 - \delta_1)([S^*] + [DS^*]) \implies Q = \delta_1[S]_T + (\delta_2 - \delta_1)([S^*] + [DS^*])$$
$$\implies [S]_T \approx \frac{Q}{\delta_1} + \left(1 - \frac{\delta_2}{\delta_1}\right)[S^*]$$

For purposes of display, the case of equal K_Ms (i.e. $K_{M,M} = K_{M,D}$) is analyzed below. Analyses of scenarios with substantially different K_Ms are left to future work. It follows that $K_{M,1} \approx K_{M,2}$ because $k_{+,M}, k_{+,D} \gg \delta_1, \delta_2$ for the majority of enzymes.

1.3.4 Parameter Values

The following figure illustrates the biological relevance of our choices of parameter values. The curve in each plot is a kernel density estimate of the experimental values obtained from the *BRENDA* enzyme database (1).

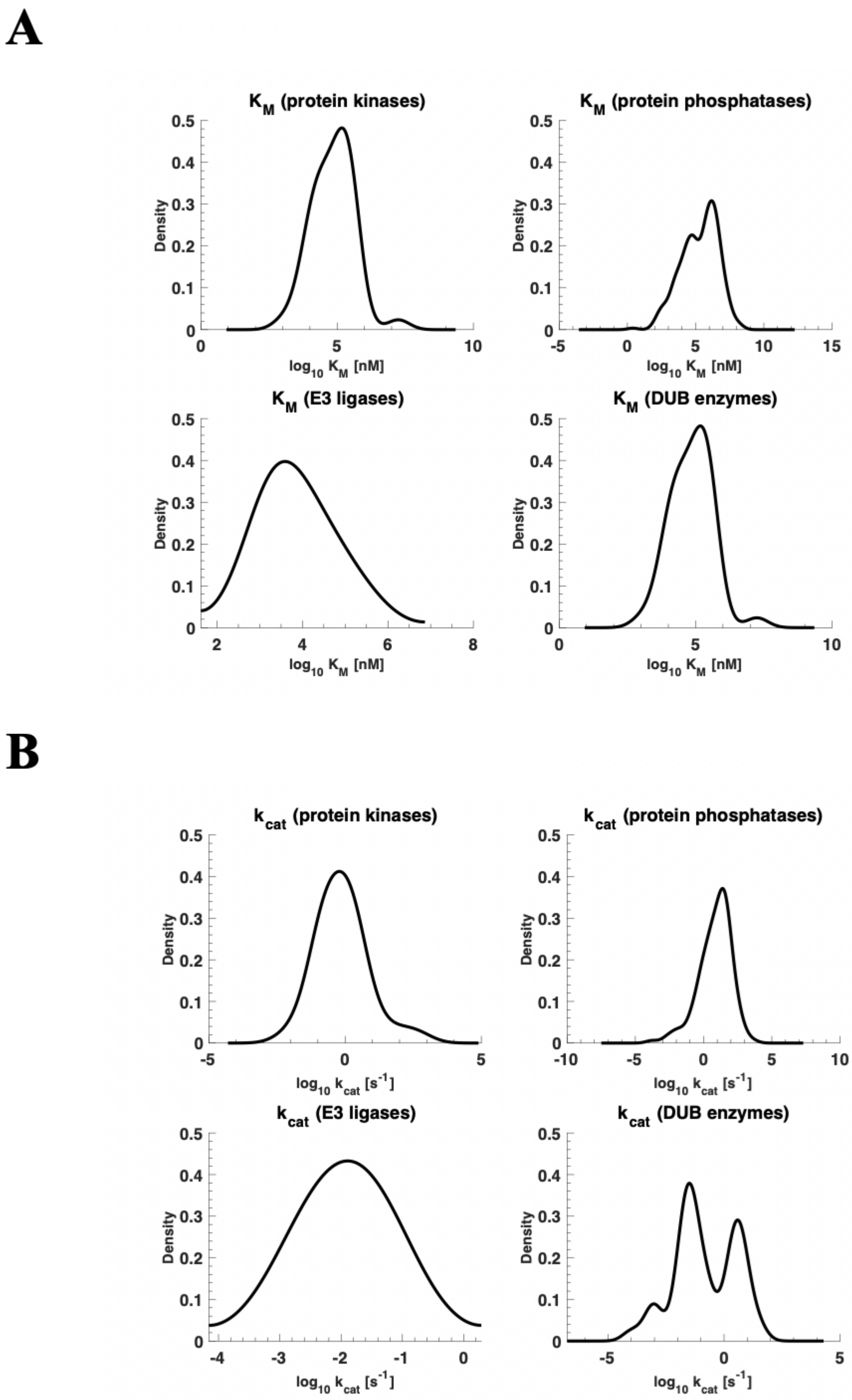

Figure S1: **Distributions of relevant steady-state kinetic parameters.** (A) Logarithmic-scale density plots of the K_Ms for protein kinases, protein phosphatases, E3 ligases, and DUB enzymes in the human genome. Experimental values were obtained from the *BRENDA* enzyme database (1) by using EC numbers for the tyrosine kinases/phosphatases and serine/threonine kinases/phosphatases. (B) Identical procedure as in the previous panel, implemented for k_{cat}s.

1.4 Analytical expressions for r_{50}

Let r denote the ratio of maximum velocities of the two enzymes (i.e. $r \equiv \frac{V_{max,M}}{V_{max,D}} = \frac{k_{cat,M}[M]_T}{k_{cat,D}[D]_T}$). This ratio represents the signal source for the system as defined previously (2). Let $\alpha \equiv [S^*]/[S]_T$, or the molar fraction of modified substrate at steady-state. The r_{50} of the response is defined as the amount of r necessary to yield a 50% response in modified substrate, or $\alpha = 0.5$.

We can rewrite eq. (8) by replacing $[MS]$ and $[DS^*]$ with their Michaelis-Menten forms. We can then divide through by $k_{cat,D}[D]_T$ and the resulting equation can be solved for r:

$$\begin{aligned}
\frac{k_{cat,M}[M]_T[S]}{K_M+[S]} - \delta_2[S^*] &= [S^*]\left(\frac{k_{cat,D}[D]_T}{K_M+[S^*]} + \frac{\delta_2[D]_T}{K_M+[S^*]}\right) \\
\frac{k_{cat,M}[M]_T[S]}{k_{cat,D}[D]_T(K_M+[S])} - \frac{\delta_2[S^*]}{k_{cat,D}[D]_T} &= [S^*]\left(\frac{1}{K_M+[S^*]} + \frac{\delta_2[D]_T}{(k_{cat,D}[D]_T)(K_M+[S^*])}\right) \\
\frac{r[S]}{K_M+[S]} &= \frac{[S^*]}{k_{cat,D}}\left(\frac{\delta_2}{[D]_T} + \frac{\delta_2+k_{cat,D}}{K_M+[S^*]}\right) \\
r &= \frac{[S^*]}{k_{cat,D}}\left(\frac{\delta_2}{[D]_T} + \frac{\delta_2+k_{cat,D}}{K_M+[S^*]}\right)\left(1+\frac{K_M}{[S]}\right) \qquad (10)
\end{aligned}$$

1.4.1 Intermediate Model

For the intermediate model, since $Q, \delta_1 > 0$, note that eq. (10) becomes:

$$\begin{aligned}
r &= \frac{\alpha}{k_{cat,D}}\left(\frac{\delta_1[S]_T}{[D]_T} + \frac{(\delta_1+k_{cat,D})[S]_T}{K_M+[S^*]}\right)\left(1+\frac{K_M}{(1-\alpha)[S]_T}\right) \\
&= \frac{\alpha}{k_{cat,D}}\left(\frac{Q}{[D]_T} + \frac{Q(\delta_1+k_{cat,D})}{\delta_1 K_M+\delta_1[S^*]}\right)\left(1+\frac{\delta_1 K_M}{(1-\alpha)Q}\right) \\
&= \frac{Q\alpha}{k_{cat,D}}\left(\frac{1}{[D]_T} + \frac{(\delta_1+k_{cat,D})}{\delta_1 K_M+\alpha Q}\right)\left(1+\frac{\delta_1 K_M}{(1-\alpha)Q}\right) \qquad (11)
\end{aligned}$$

We can substitute $\alpha = 0.5$ in the expression above to obtain r_{50}. Upon simplification, we obtain

$$r_{50} = 1 + \frac{Q}{2k_{cat,D}[D]_T} + \frac{\delta_1(K_M+[D]_T)}{k_{cat,D}[D]_T} \qquad (12)$$

1.4.2 Full Model

For the full model, we directly solve for $[S], [S^*]$ in terms of α. Observe that $[S^*] = \alpha[S]_T = \frac{\alpha Q}{\delta_1} + \alpha[S^*](1-\frac{\delta_2}{\delta_1})$. Solving for $[S^*]$ we get

$$[S^*] = \frac{\alpha Q}{\delta_1 + \alpha(\delta_2-\delta_1)}.$$

Since $[S] = [S]_T - [S^*] = \frac{(Q-\delta_2[S^*])}{\delta_1}$, we have

$$[S] = \frac{(1-\alpha)Q}{\delta_1 + \alpha(\delta_2 - \delta_1)}.$$

Plugging these expressions into eq. (10), we arrive at a closed-form expression for r as a function of α:

$$r = \frac{\alpha Q}{k_{cat,D}\left[\delta_1 + \alpha(\delta_2 - \delta_1)\right]}\left(\frac{\delta_2}{[D]_T} + \frac{\delta_2 + k_{cat,D}}{K_M + \frac{\alpha Q}{\delta_1 + \alpha(\delta_2 - \delta_1)}}\right)\left(1 + \frac{K_M}{\frac{(1-\alpha)Q}{\delta_1+\alpha(\delta_2-\delta_1)}}\right) \tag{13}$$

Substituting $\alpha = 0.5$ in eq. (13) and simplifying the resulting expression yields:

$$r_{50} = 1 + \frac{Q\delta_2}{k_{cat,D}[D]_T(\delta_1 + \delta_2)} + \frac{\delta_2(K_M + [D]_T)}{k_{cat,D}[D]_T} \tag{14}$$

1.5 Analytical expressions for n_{eff}

The effective Hill coefficient n_{eff} is defined in (4) as $\log(81)/\log(\frac{EC_{90}}{EC_{10}})$. From (5), we have the expression for n_{eff} in the Goldbeter-Koshland model:

$$n_{eff}(\text{GK}) = \frac{\log(81)}{\log(81) + 2\log\left(\frac{\frac{K_M}{[S]_T} + 0.1}{\frac{K_M}{[S]_T} + 0.9}\right)}$$

We can substitute $\alpha = 0.1, 0.9$ in both eq. (11) and eq. (13) to yield both EC_{10} and EC_{90} for the intermediate and full models, respectively. Simplifying, we obtain:

$$n_{eff}(\text{Intermediate}) = \frac{\log(81)}{\log(81) + 2\log\left(\frac{\frac{K_M}{[S]_T} + 0.1}{\frac{K_M}{[S]_T} + 0.9}\right) + \log\left(\frac{\frac{(k_{cat,D}+\delta_1)[D]_T}{Q} + \frac{K_M}{[S]_T} + 0.9}{\frac{(k_{cat,D}+\delta_1)[D]_T}{Q} + \frac{K_M}{[S]_T} + 0.1}\right)}$$

and

$$n_{eff}(\text{Full}) = \frac{\log(81)}{\log(81) + \log\left(\frac{x(Q + K_M x)(Q + K_M y)(\delta_2[9Q + y(K_M + [D]_T)] + [D]_T k_{cat,D} y)}{y(9Q + K_M x)(9Q + K_M y)(\delta_2[Q + x(K_M + [D]_T)] + [D]_T k_{cat,D} x)}\right)}$$

where $x = 9\delta_1 + \delta_2$ and $y = 9\delta_2 + \delta_1$.

1.6 Analysis of r_{50} in saturated regimes

We show that $r_{50}(\text{GK}) < r_{50}(\text{Intermediate}) < r_{50}(\text{Full})$ when total substrate is at saturating concentrations (i.e. when $[S]_T \gg K_M$).
First observe that $r_{50}(\text{GK}) = 1$ in a saturated regime. This can be seen by setting $\delta_2 = 0$ in eq. (10) and noting that $[S], [S^*] \gg K_M$:

$$r = \frac{[S^*]}{k_{cat,D}} \left(\frac{k_{cat,D}}{K_M + [S^*]} \right) \left(1 + \frac{K_M}{[S]} \right) = \frac{\frac{[S^*]}{K_M + [S^*]}}{\frac{[S]}{K_M + [S]}} = 1$$

Now

$$r_{50}(\text{GK}) < 1 + \frac{Q}{2k_{cat,D}[D]_T} + \frac{\delta_1(K_M + [D]_T)}{k_{cat,D}[D]_T} = \underbrace{1 + \frac{Q\delta_2}{k_{cat,D}[D]_T(\delta_2 + \delta_2)} + \frac{\delta_1(K_M + [D]_T)}{k_{cat,D}[D]_T}}_{r_{50}(\text{Intermediate})}$$

$$< \underbrace{1 + \frac{Q\delta_2}{k_{cat,D}[D]_T(\delta_1 + \delta_2)} + \frac{\delta_2(K_M + [D]_T)}{k_{cat,D}[D]_T}}_{r_{50}(\text{Full})}$$

Furthermore, we can show that different modes of enzyme saturation (i.e. increasing Q vs. decreasing K_M) effect substrate responses in distinct ways. Specifically, varying Q has a stronger effect on r_{50} than varying K_M - this is true in both the Intermediate and Full models. We use the following chain of (unitless) inequalities to order the quantities of interest:

$$\frac{Q\delta_2}{k_{cat,D}[D]_T(\delta_1 + \delta_2)} > \frac{Q\delta_2}{k_{cat,D}[D]_T} > \frac{K_M\delta_2}{k_{cat,D}[D]_T} > \frac{K_M\delta_1}{k_{cat,D}[D]_T}$$

which is equivalent to

$$\frac{\partial r_{50}(\text{Full})}{\partial \log Q} > \frac{\partial r_{50}(\text{Intermediate})}{\partial \log Q} > \frac{\partial r_{50}(\text{Full})}{\partial \log K_M} > \frac{\partial r_{50}(\text{Intermediate})}{\partial \log K_M}$$

The only condition on this chain is $\frac{Q}{\delta_1} > 2K_M$, which is trivially satisfied in the saturated regime (i.e. $[S]_T \gg K_M$).

1.7 Analysis of n_{eff}

When total substrate is at saturating concentrations, since $n_{eff}(\text{Intermediate})$ has an additional positive term in the denominator compared to $n_{eff}(\text{GK})$, $n_{eff}(\text{Intermediate}) < n_{eff}(\text{GK})$. Although it would be desirable to compare $n_{eff}(\text{GK})$ with $n_{eff}(\text{Full})$, this is hard to do for two reasons. Firstly, $[S]_T$ is not a function of r in the Goldbeter-Koshland model but $[S]_T$ is a function of r in the Full model. Secondly, $[S]_T$ is the solution of a cubic equation in both models, making analysis difficult.

However, we can show that in the limit $K_M \to 0, n_{eff}$(Intermediate) is a strictly decreasing function in Q:

$$n_{eff}(\text{Intermediate}) = \frac{\log(81)}{\log(81) + \log\left(\frac{\frac{(k_{cat,D}+\delta_1)[D]_T}{Q} + 0.9}{\frac{(k_{cat,D}+\delta_1)[D]_T}{Q} + 0.1}\right)}$$

has a negative partial derivative with respect to Q. Entering the expression into Mathematica (3) and simplifying yields:

$$\frac{\partial n_{eff}(\text{Intermediate})}{\partial Q} = -\frac{0.8(k_{cat,D} + \delta_1)}{(k_{cat,D} + \delta_1 + 0.1Q)(k_{cat,D} + \delta_1 + 0.9Q)\left[\log(81) + \log\left(\frac{\frac{(k_{cat,D}+\delta_1)[D]_T}{Q} + 0.9}{\frac{(k_{cat,D}+\delta_1)[D]_T}{Q} + 0.1}\right)\right]^2}$$
$$< 0$$

We can also show that n_{eff}(Intermediate) > 1 for all possible values of the parameters in the model. This is equivalent to showing

$$\left(\frac{\frac{K_M}{[S]_T} + 0.1}{\frac{K_M}{[S]_T} + 0.9}\right)^2 \left(\frac{\frac{(k_{cat,D}+\delta_1)[D]_T}{Q} + \frac{K_M}{[S]_T} + 0.9}{\frac{(k_{cat,D}+\delta_1)[D]_T}{Q} + \frac{K_M}{[S]_T} + 0.1}\right) < 1$$

or

$$\left(\frac{10\delta_1 K_M + Q}{10\delta_1 K_M + 9Q}\right)^2 \left(\frac{10(k_{cat,D} + \delta_1)[P]_T + 10\delta_1 K_M + 9Q}{10(k_{cat,D} + \delta_1)[P]_T + 10\delta_1 K_M + Q}\right) < 1$$

or

$$(10\delta_1 K_M + Q)^2(10(k_{cat,D} + \delta_1)[P]_T + 10\delta_1 K_M + 9Q) <$$
$$(10\delta_1 K_M + 9Q)^2(10(k_{cat,D} + \delta_1)[P]_T + 10\delta_1 K_M + Q)$$

We can rewrite the above inequality as follows (3):

$$8Q[(10\delta_1 K_M + Q)^2 + 8Q(10\delta_1 K_M + Q) + 2(10\delta_1 K_M + Q)(10(k_{cat,D} + \delta_1)[P]_T)$$
$$+ 8Q(10(k_{cat,D} + \delta_1)[P]_T)] > 0$$

which is true because the expression on the left-hand side is a combination of products and sums of positive terms.

1.8 Analytical expression for r_{50} of $[S]_T$ in Full model

Defining r as in the previous section (i.e. $r = \frac{k_{cat,M}[M]_T}{k_{cat,D}[D]_T}$), let $\beta \equiv [S]_T$, the amount of total substrate at steady-state. The r_{50} of the response β is defined as the amount of r necessary to yield a 50% response in total substrate. This quantity is of interest only in the full model, since β

is not constant with respect to r.

The half-maximal response for total substrate occurs at $\frac{1}{2}(\min[S]_T + \max[S]_T)$. Since

$$[S]_T = \frac{Q}{\delta_1} + \left(1 - \frac{\delta_2}{\delta_1}\right)[S^*] \tag{15}$$

we can also solve for $[S]_T$ in terms of $[S]$:

$$[S]_T = \frac{Q}{\delta_1} + \left(1 - \frac{\delta_2}{\delta_1}\right)[S^*] \implies [S]_T = \frac{Q}{\delta_1} + \left(1 - \frac{\delta_2}{\delta_1}\right)([S]_T - [S])$$

Hence

$$[S]_T = \frac{Q}{\delta_2} + \left(1 - \frac{\delta_1}{\delta_2}\right)[S] \tag{16}$$

From eq. (15), we see that $[S]_T \leq \frac{Q}{\delta_1}$ for all $[S^*] \geq 0$, since $\delta_2 > \delta_1$. Thus $\max([S]_T) = \frac{Q}{\delta_1}$ when $[S^*] = 0$. Similarly, from eq. (16), $[S]_T \geq \frac{Q}{\delta_2}$ for all $[S] \geq 0$, since $\delta_2 > \delta_1$. Thus $\min([S]_T) = \frac{Q}{\delta_2}$ when $[S] = 0$.

We can plug in and solve for $\alpha = [S^*]/[S]_T$ when $\beta = [S]_T = \frac{1}{2}(\min[S]_T + \max[S]_T)$:

$$\frac{\min[S]_T + \max[S]_T}{2} = \frac{Q}{\delta_1} + \left(1 - \frac{\delta_2}{\delta_1}\right)[S^*] \implies \frac{Q}{2}\left(\frac{1}{\delta_1} + \frac{1}{\delta_2}\right) = \frac{Q}{\delta_1} + \left(1 - \frac{\delta_2}{\delta_1}\right)[S^*] \implies [S^*] = \frac{Q}{2\delta_2}$$

$$\alpha = \frac{[S^*]}{[S]_T} = \frac{Q/(2\delta_2)}{\frac{Q}{2}(1/\delta_1 + 1/\delta_2)} = \frac{\delta_1}{\delta_1 + \delta_2}$$

Substituting $\alpha = \dfrac{\delta_1}{\delta_1 + \delta_2}$ in eq. (13) and simplifying the resulting expression yields:

$$r_{50} = \frac{Q + 2K_M\delta_1}{Q + 2K_M\delta_2}\left(1 + \frac{Q\delta_2}{k_{cat,D}[D]_T(\delta_2 + \delta_2)} + \frac{\delta_2(K_M + [D]_T)}{k_{cat,D}[D]_T}\right) \tag{17}$$

1.9 Analytical expression for n_{eff} of $[S]_T$ in Full model

To obtain an expression for n_{eff}, we need to first derive EC_{10} and EC_{90}. Note that the 10% response for total substrate occurs at $\frac{9}{10}\min[S]_T + \frac{1}{10}\max[S]_T$ and the 90% response for total substrate occurs at $\frac{1}{10}\min[S]_T + \frac{9}{10}\max[S]_T$.

To derive $EC_{10}(\beta)$, we can plug in and solve for α when $\beta = \frac{9}{10}\min[S]_T + \frac{1}{10}\max[S]_T$:

$$\frac{9}{10}\min[S]_T + \frac{1}{10}\max[S]_T = \frac{Q}{\delta_1} + \left(1 - \frac{\delta_2}{\delta_1}\right)[S^*] \implies \frac{9Q}{10\delta_2} + \frac{Q}{10\delta_1} = \frac{Q}{\delta_1} + \left(1 - \frac{\delta_2}{\delta_1}\right)[S^*]$$

$$\implies [S^*] = \frac{9}{10}\left(\frac{Q}{\delta_2}\right)$$

$$\alpha = \frac{[S^*]}{[S]_T} = \frac{9Q/(10\delta_2)}{\frac{Q}{10}(1/\delta_1 + 9/\delta_2)} = \frac{9\delta_1}{9\delta_1 + \delta_2}$$

Substituting $\alpha = \dfrac{9\delta_1}{9\delta_1 + \delta_2}$ in eq. (13) and simplifying the resulting expression (3) yields:

$$EC_{10} = \frac{9(Q + 10K_M\delta_1)(9Q + 10[D]_T k_{cat,D} + 10K_M\delta_2 + 10[D]_T\delta_2)}{10[D]_T k_{cat,D}(9Q + 10K_M\delta_2)}$$

Similar calculations can be done to derive EC_{90}:

$$EC_{90} = \frac{(9Q + 10K_M\delta_1)(Q + 10[D]_T k_{cat,D} + 10K_M\delta_2 + 10[D]_T\delta_2)}{90[D]_T k_{cat,D}(Q + 10K_M\delta_2)}$$

Thus $n_{eff} = \log(81)/\log\left(\dfrac{EC_{90}}{EC_{10}}\right)$

$$= \frac{\log(81)}{\log(81) + \log\left(\dfrac{(Q + 10K_M\delta_1)(Q + 10K_M\delta_2)(10k_{cat,D}[D]_T + 9Q + 10(K_M + [D]_T)\delta_2)}{(9Q + 10K_M\delta_1)(9Q + 10K_M\delta_2)(10k_{cat,D}[D]_T + Q + 10(K_M + [D]_T)\delta_2)}\right)}$$

2 Single Substrate, Multiple Modification States

We generalize the model in the previous section to various models with arbitrarily long chains of ubiquitin units. In these models, substrates with zero to three ubiquitin units attached are degraded at the first-order rate δ_1 and substrates with at least four units are degraded at the rate $\delta_2 > \delta_1$. In what follows, the maximal length of the chain is denoted by ℓ. The indices i and j represent the number of the ubiquitin unit and range from 0 to 3 and 4 to ℓ respectively. The index x ranges from 1 to 3.

2.1 Model with Distributive E3 & Trunk DUB

2.1.1 *Enzymatic reaction scheme*

$$M + S^{(0)} \underset{k_{-,M}}{\overset{k_{+,M}}{\rightleftharpoons}} MS^{(0)} \xrightarrow{k_{cat,M}} M + S^{(1)}$$

$$M + S^{(1)} \underset{k_{-,M}}{\overset{k_{+,M}}{\rightleftharpoons}} MS^{(1)} \xrightarrow{k_{cat,M}} M + S^{(2)}$$

$$\vdots$$

$$M + S^{(\ell-1)} \underset{k_{-,M}}{\overset{k_{+,M}}{\rightleftharpoons}} MS^{(\ell-1)} \xrightarrow{k_{cat,M}} M + S^{(\ell)}$$

$$D + S^{(1)} \underset{k_{-,D}}{\overset{k_{+,D}}{\rightleftharpoons}} DS^{(1)} \xrightarrow{k_{cat,D}} D + S^{(0)}$$

$$D + S^{(2)} \underset{k_{-,D}}{\overset{k_{+,D}}{\rightleftharpoons}} DS^{(2)} \xrightarrow{k_{cat,D}} D + S^{(0)}$$

$$\vdots$$

$$D + S^{(\ell)} \underset{k_{-,D}}{\overset{k_{+,D}}{\rightleftharpoons}} DS^{(\ell)} \xrightarrow{k_{cat,D}} D + S^{(0)}$$

$$\emptyset \xrightarrow{Q} S^{(0)}$$

$$S^{(i)} \xrightarrow{\delta_1} \emptyset$$

$$S^{(j)} \xrightarrow{\delta_2} \emptyset$$

$$MS^{(i)} \xrightarrow{\delta_1} M$$

$$MS^{(j)} \xrightarrow{\delta_2} M$$

$$DS^{(x)} \xrightarrow{\delta_1} D$$

$$DS^{(j)} \xrightarrow{\delta_2} D$$

2.1.2 Parameter Values

The values for parameters in the table below were chosen to be consistent with those of the single substrate models. The parameters listed here correspond to the deterministic version of the models. However, $[S]_T$ is one order of magnitude smaller. Thus, the corresponding values for $Q, k_{+,M}$ and $k_{+,D}$ are also scaled accordingly (i.e. Q is scaled down by a factor of ten, while $k_{+,M}$ and $k_{+,D}$ are scaled up by the same factor). Our motivation for this choice of $[S]_T$ was to lower the computational cost associated with the stochastic agent-based simulations, effectively decreasing the total number of agents in these simulations (see Section 2.8).

Parameter	Value	Units
$\boldsymbol{Q}$(unsat.)	2.00×10^{-3}	$[nM] \cdot [s]^{-1}$
$\boldsymbol{Q}$(sat.)	2.00×10^{-1}	$[nM] \cdot [s]^{-1}$
$\boldsymbol{k_{+,M}}$	1.00×10^{-3}	$[nM \cdot s]^{-1}$
$\boldsymbol{k_{+,D}}$	1.00×10^{-3}	$[nM \cdot s]^{-1}$
$\boldsymbol{k_{-,M}}$	1.00×10^{-3}	$[s]^{-1}$
$\boldsymbol{k_{-,D}}$	1.00×10^{-3}	$[s]^{-1}$
$\boldsymbol{k_{cat,M}}$	9.99×10^{-1}	$[s]^{-1}$
$\boldsymbol{k_{cat,D}}$	9.99×10^{-1}	$[s]^{-1}$
$\boldsymbol{\delta_1}$	2.00×10^{-5}	$[s]^{-1}$
$\boldsymbol{\delta_2}$	2.00×10^{-4}	$[s]^{-1}$

2.2 Model with Distributive E3 & Distributive/Sequential DUB

2.2.1 Enzymatic reaction scheme

$$M + S^{(0)} \underset{k_{-,M}}{\overset{k_{+,M}}{\rightleftharpoons}} MS^{(0)} \xrightarrow{k_{cat,M}} M + S^{(1)}$$

$$M + S^{(1)} \underset{k_{-,M}}{\overset{k_{+,M}}{\rightleftharpoons}} MS^{(1)} \xrightarrow{k_{cat,M}} M + S^{(2)}$$

$$\vdots$$

$$M + S^{(\ell-1)} \underset{k_{-,M}}{\overset{k_{+,M}}{\rightleftharpoons}} MS^{(\ell-1)} \xrightarrow{k_{cat,M}} M + S^{(\ell)}$$

$$D + S^{(1)} \underset{k_{-,D}}{\overset{k_{+,D}}{\rightleftharpoons}} DS^{(1)} \xrightarrow{k_{cat,D}} D + S^{(0)}$$

$$D + S^{(2)} \underset{k_{-,D}}{\overset{k_{+,D}}{\rightleftharpoons}} DS^{(2)} \xrightarrow{k_{cat,D}} D + S^{(1)}$$

$$\vdots$$

$$D + S^{(\ell)} \underset{k_{-,D}}{\overset{k_{+,D}}{\rightleftharpoons}} DS^{(\ell)} \xrightarrow{k_{cat,D}} D + S^{(\ell-1)}$$

$$\emptyset \xrightarrow{Q} S^{(0)}$$

$$S^{(i)} \xrightarrow{\delta_1} \emptyset$$

$$S^{(j)} \xrightarrow{\delta_2} \emptyset$$

$$MS^{(i)} \xrightarrow{\delta_1} M$$

$$MS^{(j)} \xrightarrow{\delta_2} M$$

$$DS^{(x)} \xrightarrow{\delta_1} D$$

$$DS^{(j)} \xrightarrow{\delta_2} D$$

2.2.2 Parameter Values

The same reasoning applies here as in Section 2.1.2. In particular, there is no change in the parameterization or corresponding parameter values used for either model.

Parameter	Value	Units
$\boldsymbol{Q}$(unsat.)	2.00×10^{-3}	$[nM] \cdot [s]^{-1}$
$\boldsymbol{Q}$(sat.)	2.00×10^{-1}	$[nM] \cdot [s]^{-1}$
$\boldsymbol{k_{+,M}}$	1.00×10^{-3}	$[nM \cdot s]^{-1}$
$\boldsymbol{k_{+,D}}$	1.00×10^{-3}	$[nM \cdot s]^{-1}$
$\boldsymbol{k_{-,M}}$	1.00×10^{-3}	$[s]^{-1}$
$\boldsymbol{k_{-,D}}$	1.00×10^{-3}	$[s]^{-1}$
$\boldsymbol{k_{cat,M}}$	9.99×10^{-1}	$[s]^{-1}$
$\boldsymbol{k_{cat,D}}$	9.99×10^{-1}	$[s]^{-1}$
$\boldsymbol{\delta_1}$	2.00×10^{-5}	$[s]^{-1}$
$\boldsymbol{\delta_2}$	2.00×10^{-4}	$[s]^{-1}$

2.3 Model with Distributive E3 & Processive/Sequential DUB

2.3.1 Enzymatic reaction scheme

$$M + S^{(0)} \underset{k_{-,M}}{\overset{k_{+,M}}{\rightleftharpoons}} MS^{(0)} \xrightarrow{k_{cat,M}} M + S^{(1)}$$

$$M + S^{(1)} \underset{k_{-,M}}{\overset{k_{+,M}}{\rightleftharpoons}} MS^{(1)} \xrightarrow{k_{cat,M}} M + S^{(2)}$$

$$\vdots$$

$$M + S^{(\ell-1)} \underset{k_{-,M}}{\overset{k_{+,M}}{\rightleftharpoons}} MS^{(\ell-1)} \xrightarrow{k_{cat,M}} M + S^{(\ell)}$$

$$D + S^{(1)} \underset{k_{-,D}}{\overset{k_{+,D}}{\rightleftharpoons}} DS^{(1)} \xrightarrow{k_{cat,D}} D + S^{(0)}$$

$$D + S^{(2)} \underset{k_{-,D}}{\overset{k_{+,D}}{\rightleftharpoons}} DS^{(2)} \xrightarrow{k_{cat,D}} DS^{(1)}$$

$$\vdots$$

$$D + S^{(\ell)} \underset{k_{-,D}}{\overset{k_{+,D}}{\rightleftharpoons}} DS^{(\ell)} \xrightarrow{k_{cat,D}} DS^{(\ell-1)}$$

$$\emptyset \xrightarrow{Q} S^{(0)}$$

$$S^{(i)} \xrightarrow{\delta_1} \emptyset$$

$$S^{(j)} \xrightarrow{\delta_2} \emptyset$$

$$MS^{(i)} \xrightarrow{\delta_1} M$$

$$MS^{(j)} \xrightarrow{\delta_2} M$$

$$DS^{(x)} \xrightarrow{\delta_1} D$$

$$DS^{(j)} \xrightarrow{\delta_2} D$$

2.3.2 Parameter Values

In this model version, the changes from the previous models are reflected in a lower association rate $k_{+,D}$ and higher dissociation rate $k_{-,D}$ for the D enzyme. This parameterization is consistent with both the mechanism of D as well as the experimentally observed values in (6).

Parameter	Value	Units
$\boldsymbol{Q}$(unsat.)	2.00×10^{-3}	$[nM] \cdot [s]^{-1}$
$\boldsymbol{Q}$(sat.)	2.00×10^{-1}	$[nM] \cdot [s]^{-1}$
$\boldsymbol{k_{+,M}}$	1.00×10^{-3}	$[nM \cdot s]^{-1}$
$\boldsymbol{k_{+,D}}$	1.00×10^{-4}	$[nM \cdot s]^{-1}$
$\boldsymbol{k_{-,M}}$	1.00×10^{-3}	$[s]^{-1}$
$\boldsymbol{k_{-,D}}$	1.00×10^{-1}	$[s]^{-1}$
$\boldsymbol{k_{cat,M}}$	9.99×10^{-1}	$[s]^{-1}$
$\boldsymbol{k_{cat,D}}$	9.99×10^{-1}	$[s]^{-1}$
$\boldsymbol{\delta_1}$	2.00×10^{-5}	$[s]^{-1}$
$\boldsymbol{\delta_2}$	2.00×10^{-4}	$[s]^{-1}$

2.4 Model with Processive E3 & Trunk DUB

2.4.1 Enzymatic reaction scheme

$$M + S^{(0)} \underset{k_{-,M}}{\overset{k_{+,M}}{\rightleftharpoons}} MS^{(0)} \xrightarrow{k_{cat,1,M}} MS^{(1)}$$

$$M + S^{(1)} \underset{k_{-,M}}{\overset{k_{+,M}}{\rightleftharpoons}} MS^{(1)} \xrightarrow{k_{cat,2,M}} MS^{(2)}$$

$$\vdots$$

$$M + S^{(\ell-1)} \underset{k_{-,M}}{\overset{k_{+,M}}{\rightleftharpoons}} MS^{(\ell-1)} \xrightarrow{k_{cat,2,M}} MS^{(\ell)}$$

$$D + S^{(1)} \underset{k_{-,D}}{\overset{k_{+,D}}{\rightleftharpoons}} DS^{(1)} \xrightarrow{k_{cat,D}} D + S^{(0)}$$

$$D + S^{(2)} \underset{k_{-,D}}{\overset{k_{+,D}}{\rightleftharpoons}} DS^{(2)} \xrightarrow{k_{cat,D}} D + S^{(0)}$$

$$\vdots$$

$$D + S^{(\ell)} \underset{k_{-,D}}{\overset{k_{+,D}}{\rightleftharpoons}} DS^{(\ell)} \xrightarrow{k_{cat,D}} D + S^{(0)}$$

$$\emptyset \xrightarrow{Q} S^{(0)}$$

$$S^{(i)} \xrightarrow{\delta_1} \emptyset$$
$$S^{(j)} \xrightarrow{\delta_2} \emptyset$$
$$MS^{(i)} \xrightarrow{\delta_1} M$$
$$MS^{(j)} \xrightarrow{\delta_2} M$$
$$DS^{(x)} \xrightarrow{\delta_1} D$$
$$DS^{(j)} \xrightarrow{\delta_2} D$$

2.4.2 Parameter Values

This model variant has a parameterization similar to that of the model with Distributive E3 & Processive/Sequential DUB. However, the mechanisms of the E3 ligase and DUB enzyme are swapped here. Also, we have now introduced the parameters $k_{cat,1,M}$ and $k_{cat,2,M}$ in place of $k_{cat,M}$. Since the chemistry of an E3 ligase binding a ubiquitin unit to a protein is fundamentally different from that of an E3 ligase binding a unit to another ubiquitin unit, our parameterization is consistent with both the mechanism of E3 as well as the experimentally observed values in (6).

Parameter	Value	Units
$\boldsymbol{Q}$(unsat.)	2.00×10^{-3}	$[nM] \cdot [s]^{-1}$
$\boldsymbol{Q}$(sat.)	2.00×10^{-1}	$[nM] \cdot [s]^{-1}$
$\boldsymbol{k_{+,M}}$	1.00×10^{-4}	$[nM \cdot s]^{-1}$
$\boldsymbol{k_{+,D}}$	1.00×10^{-3}	$[nM \cdot s]^{-1}$
$\boldsymbol{k_{-,M}}$	1.00×10^{-1}	$[s]^{-1}$
$\boldsymbol{k_{-,D}}$	1.00×10^{-3}	$[s]^{-1}$
$\boldsymbol{k_{cat,1,M}}$	9.99×10^{-3}	$[s]^{-1}$
$\boldsymbol{k_{cat,2,M}}$	9.99×10^{-1}	$[s]^{-1}$
$\boldsymbol{k_{cat,D}}$	9.99×10^{-1}	$[s]^{-1}$
$\boldsymbol{\delta_1}$	2.00×10^{-5}	$[s]^{-1}$
$\boldsymbol{\delta_2}$	2.00×10^{-4}	$[s]^{-1}$

2.5 Model with Processive E3 & Distributive/Sequential DUB

2.5.1 Enzymatic reaction scheme

$$M + S^{(0)} \underset{k_{-,M}}{\overset{k_{+,M}}{\rightleftharpoons}} MS^{(0)} \xrightarrow{k_{cat,1,M}} MS^{(1)}$$

$$M + S^{(1)} \underset{k_{-,M}}{\overset{k_{+,M}}{\rightleftharpoons}} MS^{(1)} \xrightarrow{k_{cat,2,M}} MS^{(2)}$$

$$\vdots$$

$$M + S^{(\ell-1)} \underset{k_{-,M}}{\overset{k_{+,M}}{\rightleftharpoons}} MS^{(\ell-1)} \xrightarrow{k_{cat,2,M}} MS^{(\ell)}$$

$$D + S^{(1)} \underset{k_{-,D}}{\overset{k_{+,D}}{\rightleftharpoons}} DS^{(1)} \xrightarrow{k_{cat,D}} D + S^{(0)}$$

$$D + S^{(2)} \underset{k_{-,D}}{\overset{k_{+,D}}{\rightleftharpoons}} DS^{(2)} \xrightarrow{k_{cat,D}} D + S^{(1)}$$

$$\vdots$$

$$D + S^{(\ell)} \underset{k_{-,D}}{\overset{k_{+,D}}{\rightleftharpoons}} DS^{(\ell)} \xrightarrow{k_{cat,D}} D + S^{(\ell-1)}$$

$$\emptyset \xrightarrow{Q} S^{(0)}$$

$$S^{(i)} \xrightarrow{\delta_1} \emptyset$$

$$S^{(j)} \xrightarrow{\delta_2} \emptyset$$

$$MS^{(i)} \xrightarrow{\delta_1} M$$

$$MS^{(j)} \xrightarrow{\delta_2} M$$

$$DS^{(x)} \xrightarrow{\delta_1} D$$

$$DS^{(j)} \xrightarrow{\delta_2} D$$

2.5.2 Parameter Values

See the previous model variants for similar justification.

**Parameter**	**Value**	**Units**
$\boldsymbol{Q}$(unsat.)	2.00×10^{-3}	$[nM] \cdot [s]^{-1}$
$\boldsymbol{Q}$(sat.)	2.00×10^{-1}	$[nM] \cdot [s]^{-1}$
$\boldsymbol{k_{+,M}}$	1.00×10^{-4}	$[nM \cdot s]^{-1}$
$\boldsymbol{k_{+,D}}$	1.00×10^{-3}	$[nM \cdot s]^{-1}$
$\boldsymbol{k_{-,M}}$	1.00×10^{-1}	$[s]^{-1}$
$\boldsymbol{k_{-,D}}$	1.00×10^{-3}	$[s]^{-1}$
$\boldsymbol{k_{cat,1,M}}$	9.99×10^{-3}	$[s]^{-1}$
$\boldsymbol{k_{cat,2,M}}$	9.99×10^{-1}	$[s]^{-1}$
$\boldsymbol{k_{cat,D}}$	9.99×10^{-1}	$[s]^{-1}$
$\boldsymbol{\delta_1}$	2.00×10^{-5}	$[s]^{-1}$
$\boldsymbol{\delta_2}$	2.00×10^{-4}	$[s]^{-1}$

2.6 Model with Processive E3 & Processive/Sequential DUB

2.6.1 Enzymatic reaction scheme

$$M + S^{(0)} \underset{k_{-,M}}{\overset{k_{+,M}}{\rightleftharpoons}} MS^{(0)} \xrightarrow{k_{cat,1,M}} MS^{(1)}$$

$$M + S^{(1)} \underset{k_{-,M}}{\overset{k_{+,M}}{\rightleftharpoons}} MS^{(1)} \xrightarrow{k_{cat,2,M}} MS^{(2)}$$

$$\vdots$$

$$M + S^{(\ell-1)} \underset{k_{-,M}}{\overset{k_{+,M}}{\rightleftharpoons}} MS^{(\ell-1)} \xrightarrow{k_{cat,2,M}} MS^{(\ell)}$$

$$D + S^{(1)} \underset{k_{-,D}}{\overset{k_{+,D}}{\rightleftharpoons}} DS^{(1)} \xrightarrow{k_{cat,D}} D + S^{(0)}$$

$$D + S^{(2)} \underset{k_{-,D}}{\overset{k_{+,D}}{\rightleftharpoons}} DS^{(2)} \xrightarrow{k_{cat,D}} DS^{(1)}$$

$$\vdots$$

$$D + S^{(\ell)} \underset{k_{-,D}}{\overset{k_{+,D}}{\rightleftharpoons}} DS^{(\ell)} \xrightarrow{k_{cat,D}} DS^{(\ell-1)}$$

$$\emptyset \xrightarrow{Q} S^{(0)}$$

$$S^{(i)} \xrightarrow{\delta_1} \emptyset$$

$$S^{(j)} \xrightarrow{\delta_2} \emptyset$$

$$MS^{(i)} \xrightarrow{\delta_1} M$$

$$MS^{(j)} \xrightarrow{\delta_2} M$$

$$DS^{(x)} \xrightarrow{\delta_1} D$$

$$DS^{(j)} \xrightarrow{\delta_2} D$$

2.6.2 Parameter Values

See the previous model variants for similar justification.

Parameter	Value	Units
$\boldsymbol{Q}$(unsat.)	2.00×10^{-3}	$[nM] \cdot [s]^{-1}$
$\boldsymbol{Q}$(sat.)	2.00×10^{-1}	$[nM] \cdot [s]^{-1}$
$\boldsymbol{k_{+,M}}$	1.00×10^{-4}	$[nM \cdot s]^{-1}$
$\boldsymbol{k_{+,D}}$	1.00×10^{-4}	$[nM \cdot s]^{-1}$
$\boldsymbol{k_{-,M}}$	1.00×10^{-1}	$[s]^{-1}$
$\boldsymbol{k_{-,D}}$	1.00×10^{-1}	$[s]^{-1}$
$\boldsymbol{k_{cat,1,M}}$	9.99×10^{-3}	$[s]^{-1}$
$\boldsymbol{k_{cat,2,M}}$	9.99×10^{-1}	$[s]^{-1}$
$\boldsymbol{k_{cat,D}}$	9.99×10^{-1}	$[s]^{-1}$
$\boldsymbol{\delta_1}$	2.00×10^{-5}	$[s]^{-1}$
$\boldsymbol{\delta_2}$	2.00×10^{-4}	$[s]^{-1}$

2.7 Graphical results

In the following figures, in order to choose a reasonable value for ℓ, we first ran simulations such that changes in the r_{50} and n_{eff} were negligible beyond a point. Using these results, we then chose $\ell = 50$ by inspection. The full range of the transitions in the curves are visible for each model. The continuous lines indicate the numerically integrated deterministic solutions.

Note that the case corresponding to the Processive E3 & Distributive/Sequential DUB model has been presented in the Main Text.

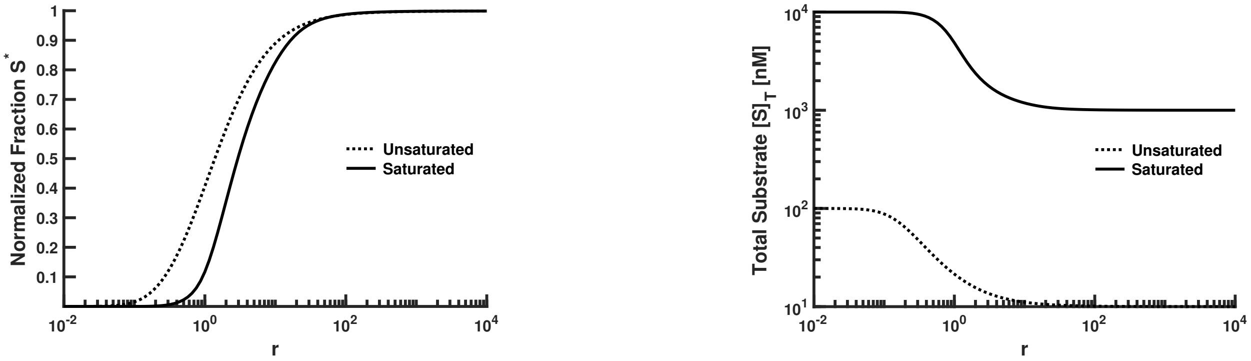

Figure S2: **Model with Distributive E3 & Trunk DUB.**

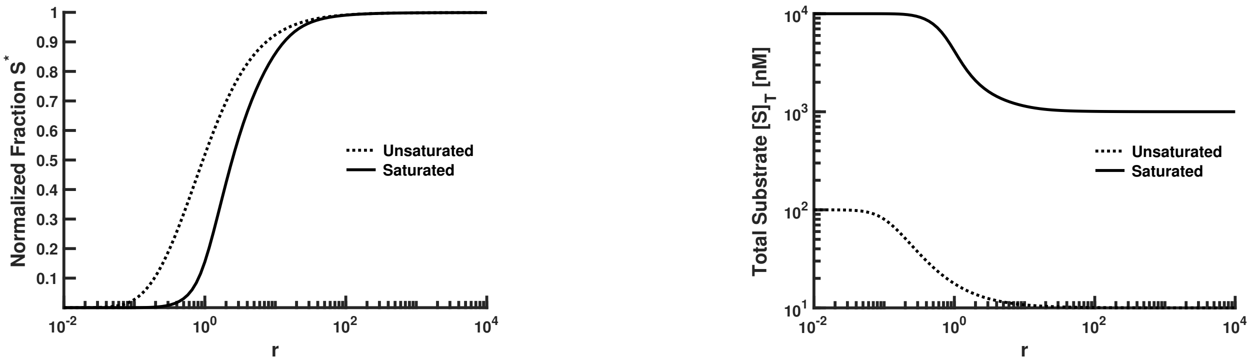

Figure S3: **Model with Distributive E3 & Distributive/Sequential DUB.**

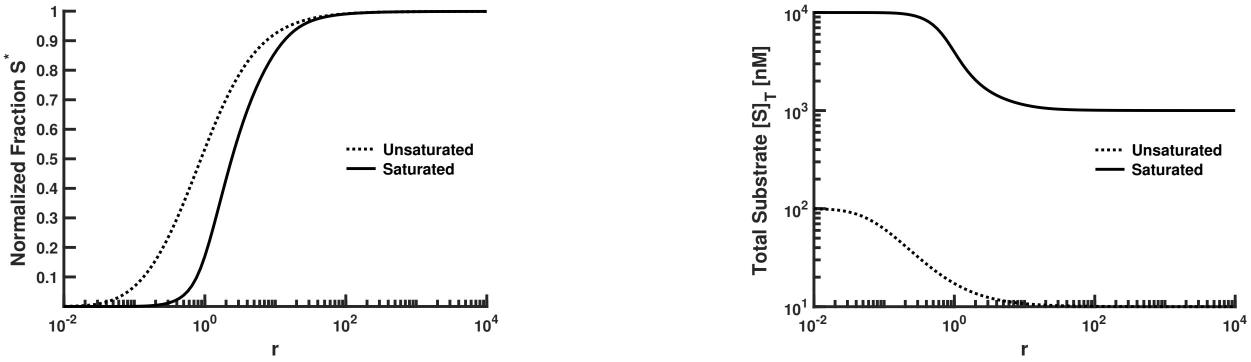

Figure S4: **Model with Distributive E3 & Processive/Sequential DUB.**

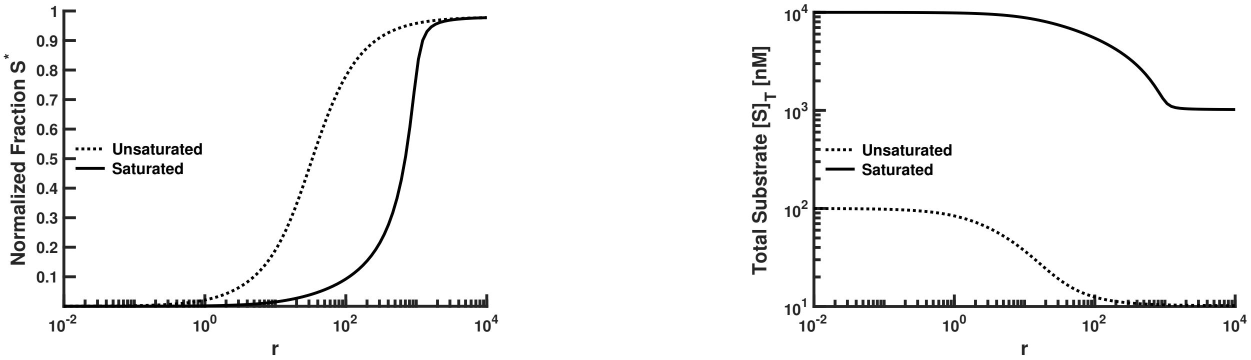

Figure S5: **Model with Processive E3 & Trunk DUB.**

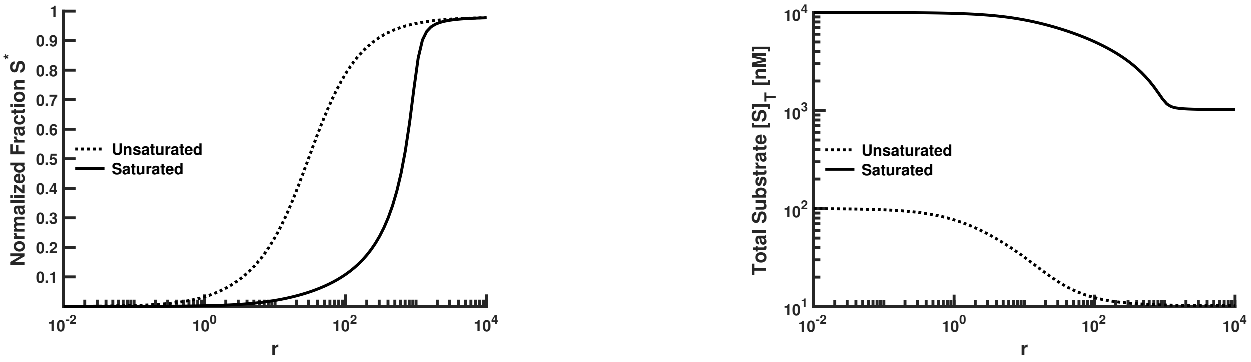

Figure S6: **Model with Processive E3 & Processive/Sequential DUB.**

2.8 Stochastic simulations

Note that the definition of a maximum length ℓ above is necessary in order to ensure a finite set of chemical reactions and ODEs. To determine if this truncation has any effect on the results, we compared our deterministic case to stochastic simulations in which we allow the ubiquitin chains to reach an arbitrary length. Our approach to developing these simulations is inspired by "agent-based" simulators developed for the stochastic simulation of rule-based models (7; 8). Due to technical considerations with the mechanism, however, we wrote our own dedicated code for these simulations, following closely the approach taken in our previous work on modeling length control in the bacterial Type III Secretion System (9).

Briefly, our simulations contain three types of agents: the M enzyme, the D enzyme, and the substrate S. These agents are represented *independently*; in other words, if there are 1000 S molecules in the simulation, this is represented by having 1000 distinct "S" agents in memory. Each S agent has associated with it a number that represents the length of its ubiquitin chain. These lengths can range from zero to the largest number that can be represented by the particular data structure. Since this number is much, much larger than the largest value ever practically observed in the simulation, this essentially corresponds to allowing for arbitrary chain lengths.

We wrote a separate simulation in C++ for all of the scenarios described above. All of the parameters from the deterministic simulations were converted to their corresponding stochastic values in a straightforward way (10). In particular, we specified a "compartment" volume of 100 fL for each model. Since these simulations are relatively expensive computationally, we performed simulations for a subset of the parameters considered in our deterministic simulations (see below). All simulations were performed until the system achieved a steady state. Simulation codes are available upon request.

2.8.1 *No truncation effect*

In the following set of figures, the dots correspond to averages from stochastic simulations. We conducted 100 simulations for each model.

We note that, within the parameter regimes we were able to simulate in a reasonable amount of time, there is excellent agreement between the deterministic and stochastic frameworks. In other words, there does not seem to be an effect of premature truncation in chain length for the deterministic model versions.

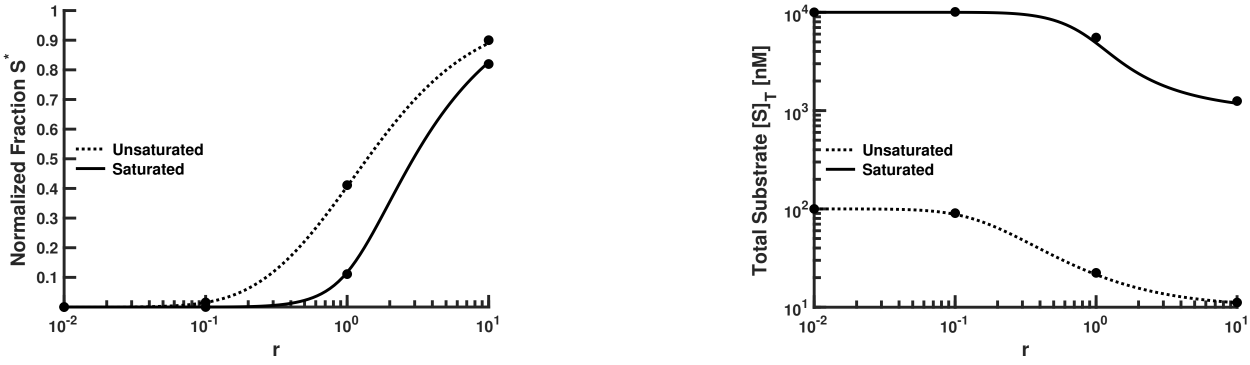

Figure S7: **Model with Distributive E3 & Trunk DUB.**

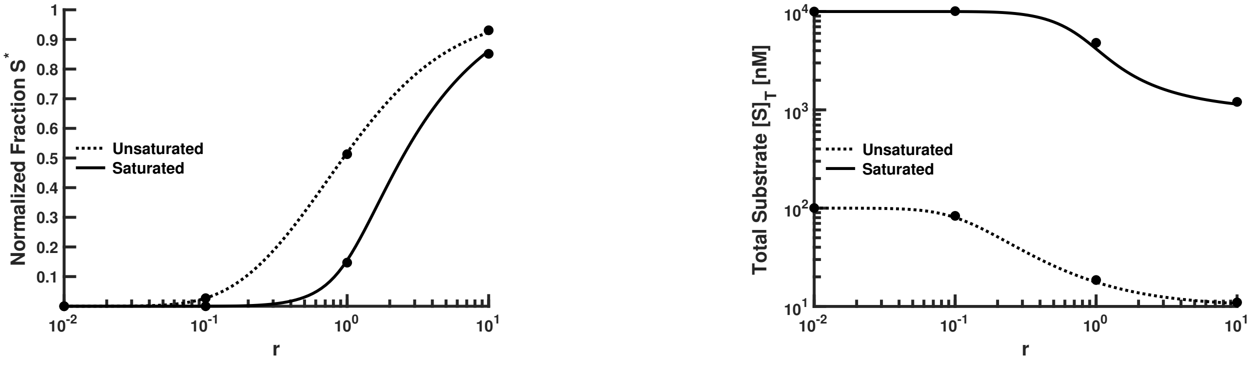

Figure S8: **Model with Distributive E3 & Distributive/Sequential DUB.**

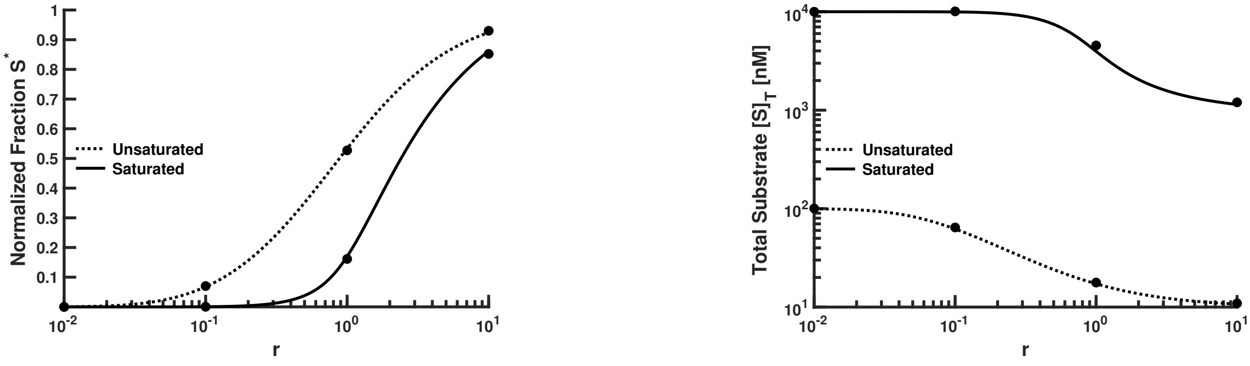

Figure S9: **Model with Distributive E3 & Processive/Sequential DUB.**

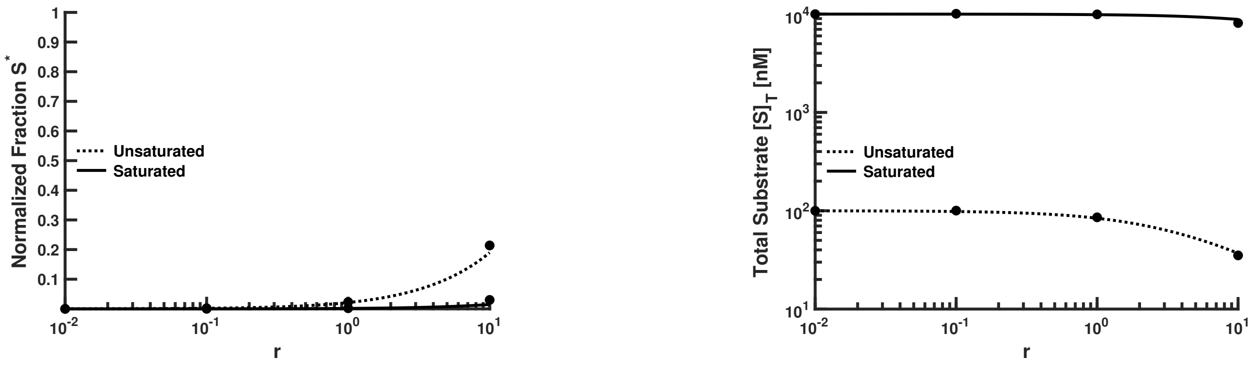

Figure S10: **Model with Processive E3 & Trunk DUB.**

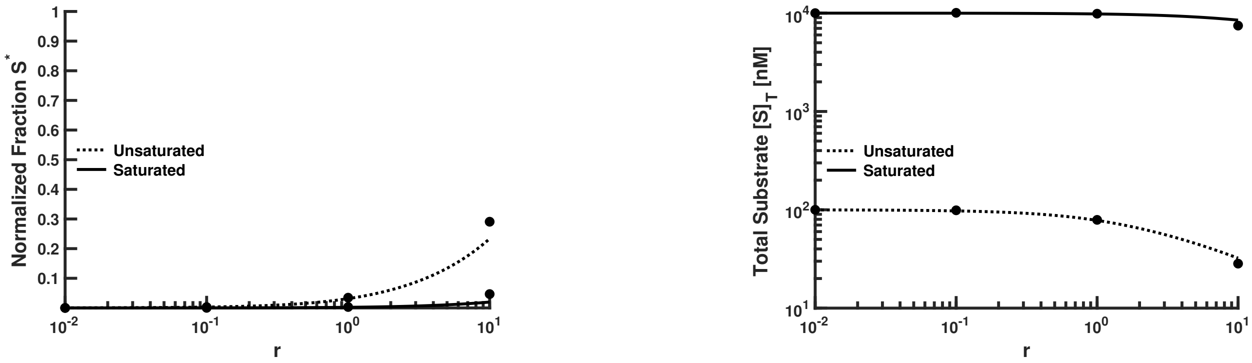

Figure S11: **Model with Processive E3 & Trunk DUB.**

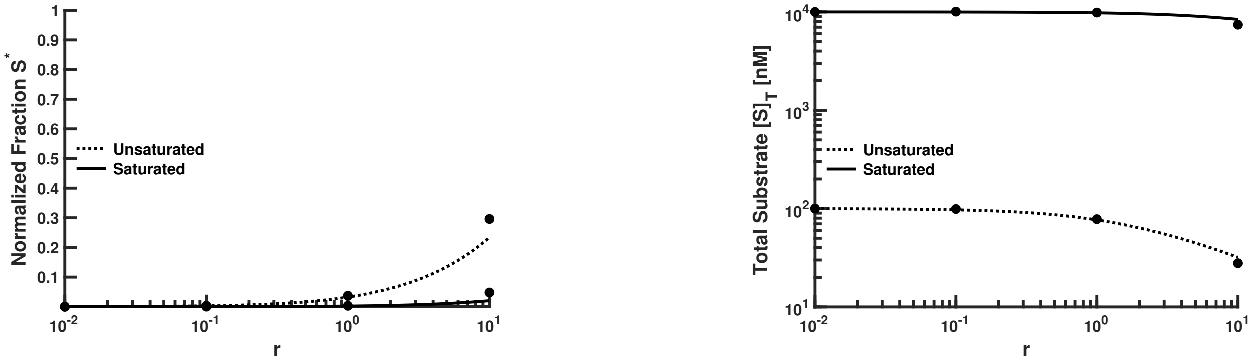

Figure S12: **Model with Processive E3 & Processive/Sequential DUB.**

2.9 Robustness of results

In order to explore the robustness of our conclusions with respect to parameter variation, we randomly sampled a reasonable region of parameter space for each of our multiple modification state models and characterized how ODE models based on those parameters behaved at steady

state. We focused on sampling different values of K_M for the enzymes and different degradation rates. To sample K_M values, we fixed the values of k_{cat} and k_- at 0.999 s^{-1} and 0.001 s^{-1}, respectively, and sampled values of k_+. We chose to do this because all of our analysis is focused on steady-state responses, and so the specific values of the rate constant only influence our results through the ratio $K_M = (k_{cat} + k_-)/k_+$. Setting k_{cat} and k_- to these values allows us to focus our analysis on the case of catalytically efficient enzymes. To sample values of K_M, we sampled k_+ from a log-uniform distribution ranging from 0.1× to 10× the K_M we originally chose for our analysis. We did this independently for both the M and D enzymes in the model. Similarly, we sampled δ_1 from a log-uniform distribution between 0.1× and 10× the original δ_1. We fixed δ_2 to equal $10 \times \delta_1$, since our model focuses on the case where the ubiquitin chain drives an increased rate of substrate degradation. Finally, two values of the Q parameter were chosen to correspond to the unsaturated and saturated regimes.

One of the key findings of our work is the fact that increasing saturation by increasing the production of substrate (our parameter Q) increases the ratio of E3 to DUB activity that is needed to see a transition in substrate concentration (i.e. it increases the r_{50} of the transition). To see if this held for different parameter sets, we computed the ratio $r_{50,saturated}/r_{50,unsaturated}$ for each of the parameter sets; values of this ratio greater than 1 indicate that saturation increases r_{50}. A histogram of this ratio over all our randomly sampled parameters indicates that they all indeed have an r_{50} ratio greater than 1.

Another major finding of our work is that the ultrasensitivity of the response (for both the molar fraction of modified substrate, $[S^*]/[S]_T$ and the total amount of substrate, $[S]_T$) is much less than predicted by the standard Goldbeter-Koshland model of PTM cycles. To characterize this, we calculated the ratio of the effective Hill coefficients, n_{eff} for the saturated vs. the unsaturated cases. We see that this ratio is generally around 1 to 2, and never much larger than 5, indicating that the large increase in ultrasensitivity observed for standard saturated PTM cycles is not observed for these parameter sets.

Thus, although we cannot exhaustively explore parameter space, these new results indicate that our main conclusions are likely robust to reasonable variations of the parameters.

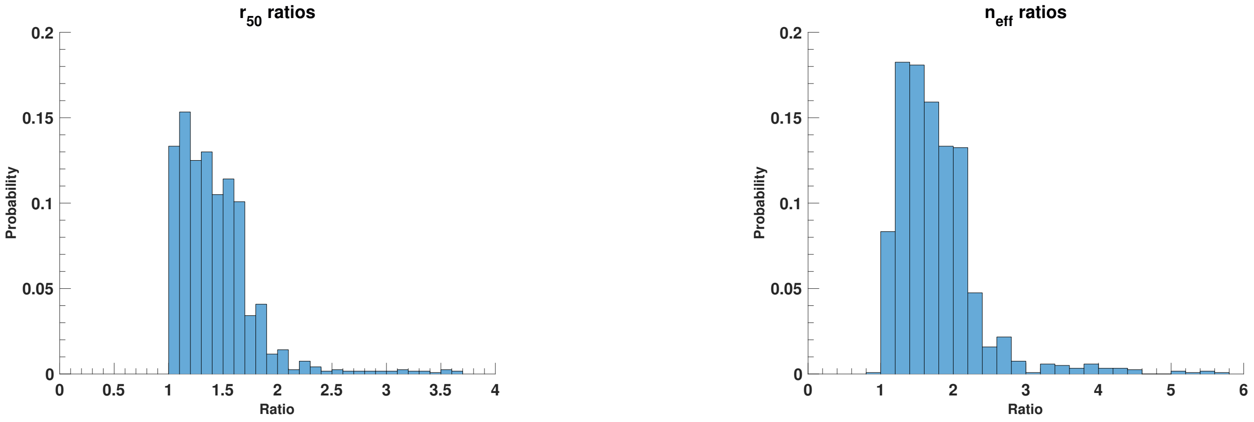

Figure S13: **Histograms of r_{50} and n_{eff} ratios.**

3 Multiple Substrates, Single Modification State

The subscript index z represents the substrate number, ranging from 1 to N, where N is the total number of substrates.

$$M + S_z \underset{k_{-,M}}{\overset{k_{+,M}}{\rightleftharpoons}} MS_z \xrightarrow{k_{cat,M}} M + S_z^*$$

$$D + S_z^* \underset{k_{-,D}}{\overset{k_{+,D}}{\rightleftharpoons}} DS_z^* \xrightarrow{k_{cat,D}} D + S_z$$

$$\emptyset \xrightarrow{Q_z} S$$

$$S_z \xrightarrow{\delta_1} \emptyset$$

$$S_z^* \xrightarrow{\delta_2} \emptyset$$

$$MS_z \xrightarrow{\delta_1} M$$

$$DS_z^* \xrightarrow{\delta_2} D$$

3.1 Equations

3.1.1 Main

$$\frac{d[S_z]}{dt} = Q_z - k_{+,M}[M][S_z] + k_{-,M}[MS_z] + k_{cat,D}[DS_z^*] - \delta_1[S_z] \tag{18a}$$

$$\frac{d[S_z^*]}{dt} = -k_{+,D}[D][S_z^*] + k_{-,D}[DS_z^*] + k_{cat,M}[MS_z] - \delta_2[S_z^*] \tag{18b}$$

$$\frac{d[MS_z]}{dt} = k_{+,M}[M][S_z] - (k_{-,M} + k_{cat,M} + \delta_1)[MS_z] \tag{18c}$$

$$\frac{d[DS_z^*]}{dt} = k_{+,D}[D][S_z^*] - (k_{-,D} + k_{cat,D} + \delta_2)[DS_z^*] \tag{18d}$$

$$\frac{d[M]}{dt} = (k_{-,M} + k_{cat,M} + \delta_1)\sum_{z=1}^{N}[MS_z] - k_{+,M}[M]\sum_{z=1}^{N}[S_z] \tag{18e}$$

$$\frac{d[D]}{dt} = (k_{-,D} + k_{cat,D} + \delta_2)\sum_{z=1}^{N}[DS_z^*] - k_{+,D}[D]\sum_{z=1}^{N}[S_z^*] \tag{18f}$$

3.1.2 Mass conservation

$$[M]_T = [M] + \sum_{z=1}^{N}[MS_z]$$

$$[D]_T = [D] + \sum_{z=1}^{N}[DS_z^*]$$

$$[S]_T = \sum_{z=1}^{N} ([S_z] + [S_z^*] + [MS_z] + [DS_z^*])$$

3.2 Comments

Adding multiple substrates to the system results in additive effects. Similar conclusions hold for multiple substrates with just one modification state as for one substrate with one modification state (Section 1). The only changes in the relevant expressions are:

(i) The substrate production rate Q becomes $\sum_z Q_z$

(ii) S becomes $\sum_z S_z$

(iii) S^* becomes $\sum_z S_z^*$

3.3 Analytical expression for $r_{50}([S_1]_T)$

By inspecting eq. (13) and its derivation, one can see that the expression for r in this case will be similar. Following the derivation in Section 1.8, we can obtain $r_{50}([S_1]_T)$ as follows:

$$r_{50}([S_1]_T) = \frac{\sum_{i=1}^{N} \frac{\alpha_i Q_i}{\delta_1 + \alpha_i(\delta_2 - \delta_1)}}{k_{cat,D}} \left(\frac{\delta_2}{[D]_T} + \frac{\delta_2 + k_{cat,D}}{K_M + \sum_{i=1}^{N} \frac{\alpha_i Q_i}{\delta_1 + \alpha_i(\delta_2 - \delta_1)}} \right) \left(1 + \frac{K_M}{\sum_{i=1}^{N} \frac{(1-\alpha_i) Q_i}{\delta_1 + \alpha_i(\delta_2 - \delta_1)}} \right)$$

where

$$\alpha_1 = \frac{\delta_1}{\delta_1 + \delta_2}$$

$$\alpha_i = EC_{100\alpha_1}\left(\frac{[S_i^*]}{[S_i]_T}\right)$$

$$N = \text{Total Number of Substrates}$$

For the sake of illustration, the figure below is for $N = 10$. The semi-analytical curve was obtained by substituting values of α_i obtained empirically from simulation into the analytical expression for $r_{50}([S_1]_T)$.

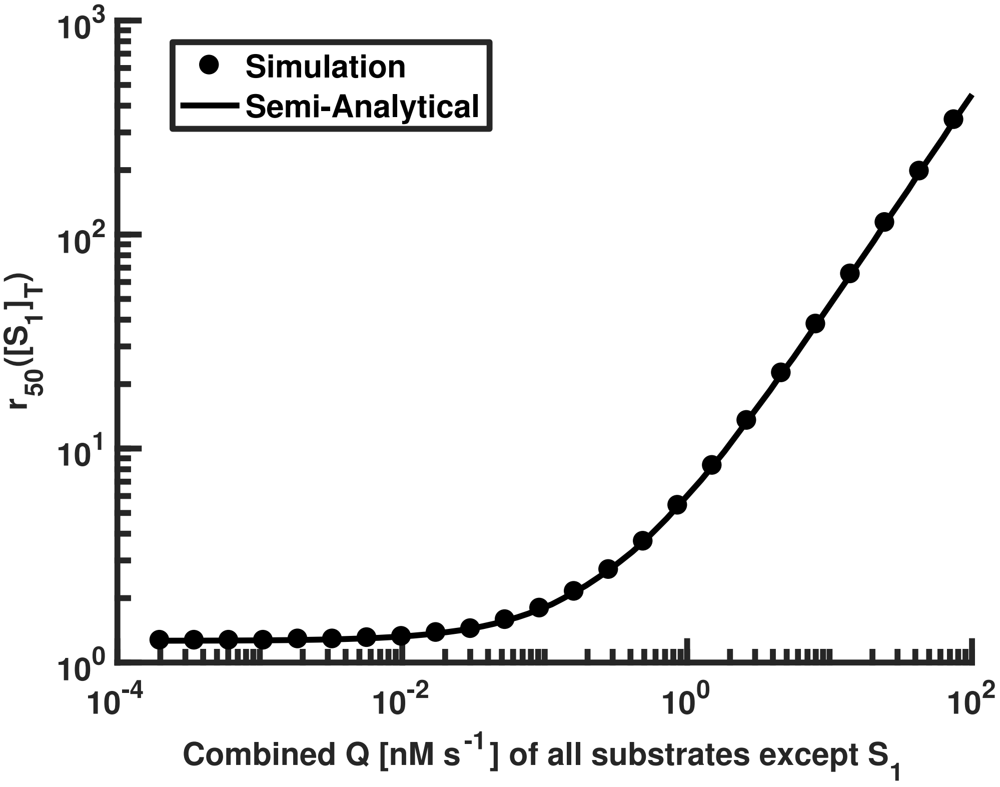

4 Multiple Substrates, Multiple Modification States

Here, we generalize the set of models with only one substrate to a set of models with multiple substrates. The parameterization of the models in this section differ from their single substrate counterparts in only one way: $N > 1$.

For the sake of illustration, the model with Processive E3 & Distributive/Sequential DUB is described by the following scheme of enzymatic reactions:

4.1 Model with Processive E3 & Distributive/Sequential DUB

$$M + S_z^{(0)} \underset{k_{-,M}}{\overset{k_{+,M}}{\rightleftharpoons}} MS_z^{(0)} \xrightarrow{k_{cat,M}} MS_z^{(1)}$$

$$M + S_z^{(1)} \underset{k_{-,M}}{\overset{k_{+,M}}{\rightleftharpoons}} MS_z^{(1)} \xrightarrow{k_{cat,M}} MS_z^{(2)}$$

$$\vdots$$

$$M + S_z^{(\ell-1)} \underset{k_{-,M}}{\overset{k_{+,M}}{\rightleftharpoons}} MS_z^{(\ell-1)} \xrightarrow{k_{cat,M}} MS_z^{(\ell)}$$

$$D + S_z^{(1)} \underset{k_{-,D}}{\overset{k_{+,D}}{\rightleftharpoons}} DS_z^{(1)} \xrightarrow{k_{cat,D}} D + S_z^{(0)}$$

$$D + S_z^{(2)} \underset{k_{-,D}}{\overset{k_{+,D}}{\rightleftharpoons}} DS_z^{(2)} \xrightarrow{k_{cat,D}} D + S_z^{(1)}$$

$$\vdots$$

$$D + S_z^{(\ell)} \underset{k_{-,D}}{\overset{k_{+,D}}{\rightleftharpoons}} DS_z^{(\ell)} \xrightarrow{k_{cat,D}} D + S_z^{(\ell-1)}$$

$$\emptyset \xrightarrow{Q} S_z^{(0)}$$

$$S_z^{(i)} \xrightarrow{\delta_1} \emptyset$$

$$S_z^{(j)} \xrightarrow{\delta_2} \emptyset$$

$$MS_z^{(i)} \xrightarrow{\delta_1} M$$

$$MS_z^{(j)} \xrightarrow{\delta_2} M$$

$$DS_z^{(x)} \xrightarrow{\delta_1} D$$

$$DS_z^{(j)} \xrightarrow{\delta_2} D$$

5 Main Text: Parameter Values

Table S1: **Fig 2C**

Parameter	Value	Units
$\boldsymbol{Q}$	1.00×10^{-1} to 1.00×10^{5}	$[nM] \cdot [s]^{-1}$
$\boldsymbol{k_+}$	1.00×10^{-4}	$[nM \cdot s]^{-1}$
$\boldsymbol{k_-}$	1.00×10^{-3}	$[s]^{-1}$
$\boldsymbol{k_{cat}}$	9.99×10^{-1}	$[s]^{-1}$
$\boldsymbol{\delta_1}$	2.00×10^{-5}	$[s]^{-1}$
$\boldsymbol{\delta_2}$	2.00×10^{-4}	$[s]^{-1}$

Table S2: **Fig 2D**

Parameter	Value	Units
$\boldsymbol{Q}$	1.00×10^{-2}	$[nM] \cdot [s]^{-1}$
$\boldsymbol{k_+}$	1.00×10^{-1} to 1.00×10^{5}	$[nM \cdot s]^{-1}$
$\boldsymbol{k_-}$	1.00×10^{-3}	$[s]^{-1}$
$\boldsymbol{k_{cat}}$	9.99×10^{-1}	$[s]^{-1}$
$\boldsymbol{\delta_1}$	2.00×10^{-5}	$[s]^{-1}$
$\boldsymbol{\delta_2}$	2.00×10^{-4}	$[s]^{-1}$

Table S3: **Fig 3A**

Parameter	Value	Units
$\boldsymbol{Q}$	2.00×10^{-2} to 2.00×10^{2}	$[nM] \cdot [s]^{-1}$
$\boldsymbol{k_+}$	1.00×10^{-4}	$[nM \cdot s]^{-1}$
$\boldsymbol{k_-}$	1.00×10^{-3}	$[s]^{-1}$
$\boldsymbol{k_{cat}}$	9.99×10^{-1}	$[s]^{-1}$
$\boldsymbol{\delta_1}$	2.00×10^{-5}	$[s]^{-1}$
$\boldsymbol{\delta_2}$	2.00×10^{-4}	$[s]^{-1}$

Table S4: **Fig 3B**

Parameter	Value	Units
$\boldsymbol{Q}$	1.00×10^{-1} to 1.00×10^{5}	$[nM] \cdot [s]^{-1}$
$\boldsymbol{k_+}$	1.00×10^{-4}	$[nM \cdot s]^{-1}$
$\boldsymbol{k_-}$	1.00×10^{-3}	$[s]^{-1}$
$\boldsymbol{k_{cat}}$	9.99×10^{-1}	$[s]^{-1}$
$\boldsymbol{\delta_1}$	2.00×10^{-5}	$[s]^{-1}$
$\boldsymbol{\delta_2}$	2.00×10^{-4}	$[s]^{-1}$

Table S5: **Fig 4A**

Parameter	Value	Units
$\boldsymbol{Q}$	1.00×10^{-2} to 1.00×10^{4}	$[nM] \cdot [s]^{-1}$
$\boldsymbol{k_+}$	1.00×10^{-4}	$[nM \cdot s]^{-1}$
$\boldsymbol{k_-}$	1.00×10^{-3}	$[s]^{-1}$
$\boldsymbol{k_{cat}}$	9.99×10^{-1}	$[s]^{-1}$
$\boldsymbol{\delta_1}$	2.00×10^{-5}	$[s]^{-1}$
$\boldsymbol{\delta_2}$	2.00×10^{-4}	$[s]^{-1}$

Table S6: **Fig 4C**

Parameter	Value	Units
$\boldsymbol{Q}$	1.00×10^{-2} to 1.00×10^{4}	$[nM] \cdot [s]^{-1}$
$\boldsymbol{k_+}$	1.00×10^{-3}	$[nM \cdot s]^{-1}$
$\boldsymbol{k_-}$	1.00×10^{-3}	$[s]^{-1}$
$\boldsymbol{k_{cat}}$	9.99×10^{-1}	$[s]^{-1}$
$\boldsymbol{\delta_1}$	2.00×10^{-5}	$[s]^{-1}$
$\boldsymbol{\delta_2}$	2.00×10^{-4}	$[s]^{-1}$

Table S7: **Fig 4D**

**Parameter**	**Value**	**Units**
$\boldsymbol{Q}$	1.00×10^{-4} to 1.00×10^{2}	$[nM] \cdot [s]^{-1}$
$\boldsymbol{k_+}$	1.00×10^{-3}	$[nM \cdot s]^{-1}$
$\boldsymbol{k_-}$	1.00×10^{-3}	$[s]^{-1}$
$\boldsymbol{k_{cat}}$	9.99×10^{-1}	$[s]^{-1}$
$\boldsymbol{\delta_1}$	2.00×10^{-5}	$[s]^{-1}$
$\boldsymbol{\delta_2}$	2.00×10^{-4}	$[s]^{-1}$

References

1. M. Scheer, *et al.*, *Nucleic Acids Res.* **39**, 670 (2011).
2. L. A. Segel, L. Edelstein-Keshet, *A Primer on Mathematical Models in Biology* (Society for Industrial and Applied Mathematics, Philadelphia, PA, 2013).
3. Wolfram Research, Inc., Mathematica 10.0.
4. W. Dubitzky, O. Wolkenhauer, H. Yokota, K.-H. Cho, *Encyclopedia of Systems Biology* (Springer, New York, NY, 2013).
5. A. Goldbeter, D. E. Koshland Jr., *PNAS* **78**, 6840 (1981).
6. N. W. Pierce, G. Kleiger, S. Shan, R. J. Deshaies, *Nature* **462**, 615 (2009).
7. D. V., F. J., F. W., K. J., *Proceedings APLAS* **4807**, 139 (2007).
8. M. W. Sneddon, J. R. Faeder, T. Emonet, *Nat Methods* **8**, 177 (2011).
9. M. K. Nariya, J. Israeli, J. J. Shi, E. J. Deeds, *PLoS Comput Biol* **12**, e1004851 (2016).
10. E. Deeds, J. Krivine, J. Feret, V. Danos, W. Fontana, *PLoS One* **7**, e32032 (2012).
